# From wireframes to immersive reality: a historical perspective on molecular visualization software

**DOI:** 10.1093/bib/bbag373

**Published:** 2026-09-07

**Authors:** Ana Lúcia Leitão, Francisco J Enguita

**Affiliations:** Departamento de Química, Faculdade de Ciências e Tecnologia, Universidade NOVA de Lisboa, 2829-516 Caparica, Portugal; Faculdade de Medicina, Universidade de Lisboa, Av. Professor Egas Moniz, 1649-028 Lisboa, Portugal

**Keywords:** molecular visualization, graphical interface, molecular dynamics, bioinformatics

## Abstract

The evolution of molecular visualization software represents a pivotal yet often underappreciated dimension of computational structural biology and bioinformatics. As the scale and complexity of structural datasets increase, spanning from cryo-electron microscopy reconstructions to proteome-wide AI-predicted models, visualization tools have become essential for data interpretation, hypothesis generation, and effective scientific communication. Widely used platforms such as PyMOL, UCSF Chimera, and emerging virtual reality environments reflect decades of progressive innovation driven by advancements in computational hardware, graphics rendering, and algorithm development. This review traces the historical development of molecular graphics software, from early vector-based applications like FRODO and O, through interactive tools including RasMol and VMD, to modern real-time 3D visualization frameworks. We highlight key technological milestones and design principles that continue to shape contemporary software architectures, underscoring the interdisciplinary collaborations among chemists, structural biologists, and computer scientists that have propelled the field. Given the current integration of artificial intelligence, immersive visualization, and cloud computing in structural bioinformatics workflows, revisiting this topic provides critical context for guiding the design of more intuitive, scalable, and accessible visualization platforms. By documenting and critically analyzing this trajectory, the present work aims to honor the foundational contributions to molecular graphics while positioning the field as a dynamic and integral component of future bioinformatics research and computational discovery.

## Introduction to molecular graphics

Molecular graphics constitute a core discipline within structural bioinformatics, providing computational frameworks to transform atomic coordinate data into multidimensional, analytically tractable representations. Since the earliest vector-based display systems, the field has integrated advances in graphical primitives, rendering algorithms, and interactive computation to enable interrogation of macromolecular architecture at atomic resolution. By converting crystallographic, magnetic resonance, or cryo-electron microscopy datasets into manipulable 3D models, molecular graphics support the identification of topological motifs, intramolecular contacts, solvent-accessible surfaces, and dynamic conformational states underlying molecular recognition and function [1].

**Figure 1:**
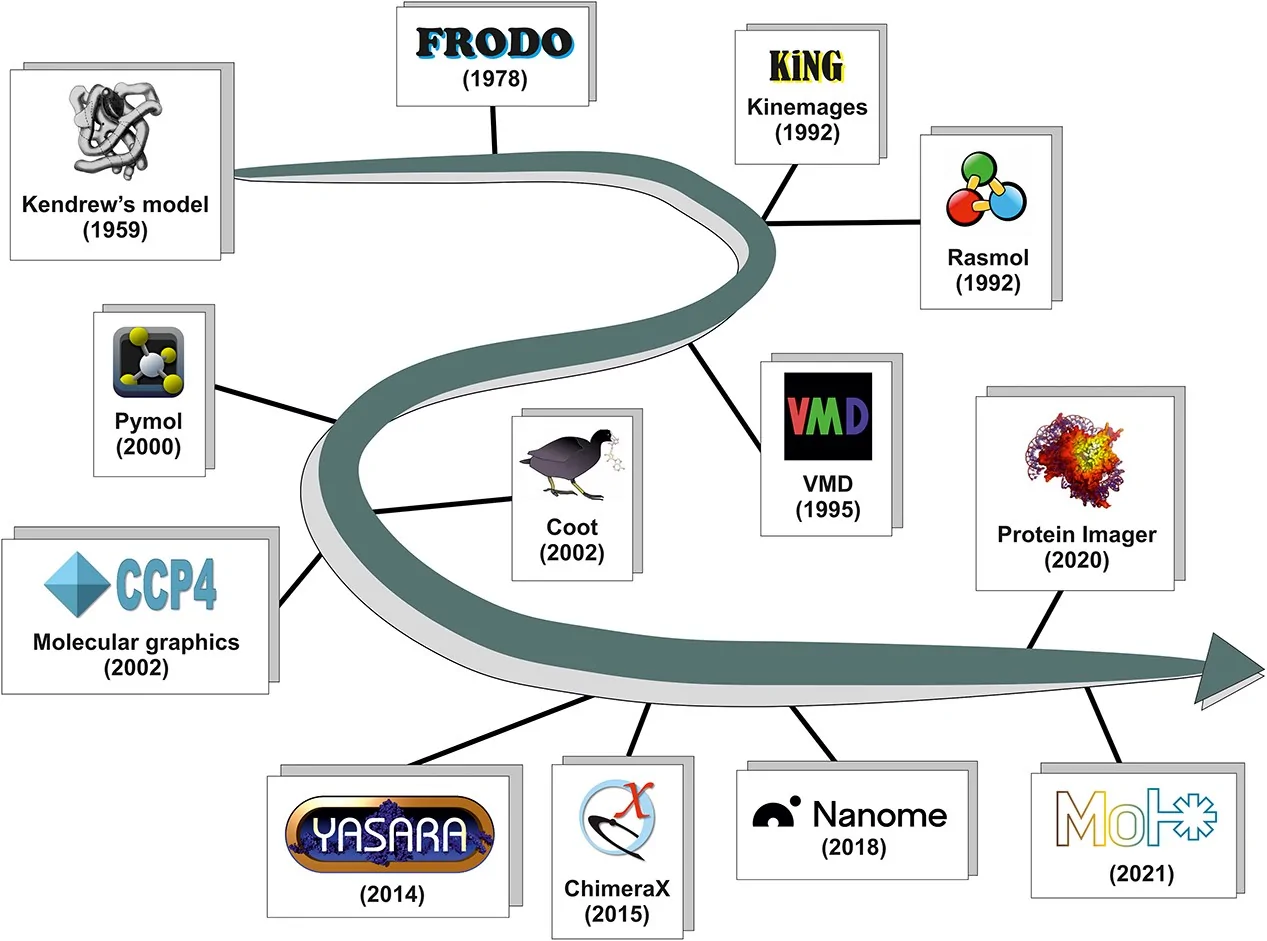
Evolution of molecular graphics tools in structural biology. Timeline illustrating key milestones in the development of molecular visualization and model-building software from the mid-20th century to the present. The first 3D atomic model by Kendrew (1959) marked the beginning of physical molecular modeling. The introduction of FRODO (1978) by Alwyn Jones enabled interactive computer-assisted fitting of atomic coordinates into electron-density maps. The early 1990s saw the emergence of Kinemages (and the KiNG viewer, 1992) and RasMol (1992), pioneering accessible digital visualization of macromolecules. Subsequent tools such as VMD (1995), PyMOL (2000), CCP4 Molecular Graphics (2002), and Coot (2002) expanded functionalities for model refinement and analysis. The later development of Yasara (2014) and ChimeraX (2015) integrated high-performance rendering and interactive analysis, while Nanome (2018), Protein Imager (2020), and MolStar (2021) represent the new generation of virtual reality and web-based visualization platforms that extend molecular graphics into immersive and collaborative environments. The arrow indicates the progressive technological evolution from mechanical models to advanced, real-time interactive visualization systems.

The advantages of computer-assisted molecular visualization over traditional mechanical and schematic models are well established [2, 3]. The deployment of interactive rendering pipelines allowed real-time movement, scaling, and stereoscopic depth perception, thereby enabling researchers to detect steric clashes, optimize conformations, and refine structural data with greater precision. Subsequent incorporation of energy minimization routines, molecular dynamics engines, and docking frameworks into visualization platforms transformed molecular graphics from a descriptive medium into an integrative analytical environment (Fig. 1). These environments now support hybrid workflows in which graphical inspection is seamlessly coupled with computational modeling, free-energy calculations, and machine-learning-driven structure prediction [4].

Modern molecular graphics systems leverage high-performance graphics processing units (GPUs), advanced shading and ray-tracing algorithms, and data-compression schemes to render complex assemblies, ranging from large ribonucleoprotein particles to entire viral capsids, in real time. The integration of immersive technologies, including virtual and augmented reality, further expands their utility in research and education by enabling intuitive exploration of high-dimensional molecular spaces. Beyond visualization, these platforms function as graphical front ends to computational pipelines, supporting hypothesis-driven analysis of conformational ensembles, ligand-binding pathways, and macromolecular assemblies (Fig. 1).

## Rods, spheres, and ingenuity

The pioneering work of John Kendrew and Max Perutz in the late 1950s established the first high-resolution protein structures, providing fundamental insights into the 3D organization of myoglobin and hemoglobin [5]. These achievements, accomplished through X-ray crystallography and Fourier synthesis, revealed for the first time the atomic architecture of globular proteins. To interpret the complex electron density maps, both investigators relied on large-scale physical models constructed from metal rods and spherical markers, which were used to represent atomic positions within the crystal lattice. Perutz’s group in Cambridge famously suspended brass rods with colored markers in Perspex scaffolds, allowing atomic coordinates derived from diffraction data to be visualized in three dimensions [6]. Although innovative, these models were cumbersome and limited: difficult to manipulate, mechanically unstable, and insufficient for depicting detailed sidechain packing or recurrent secondary-structure motifs. Kendrew’s myoglobin model, comprising nearly 2600 atoms, demanded such large-scale physical construction that appreciating the overall fold was nearly impossible without abstracting from atomic detail.

**Figure 2:**
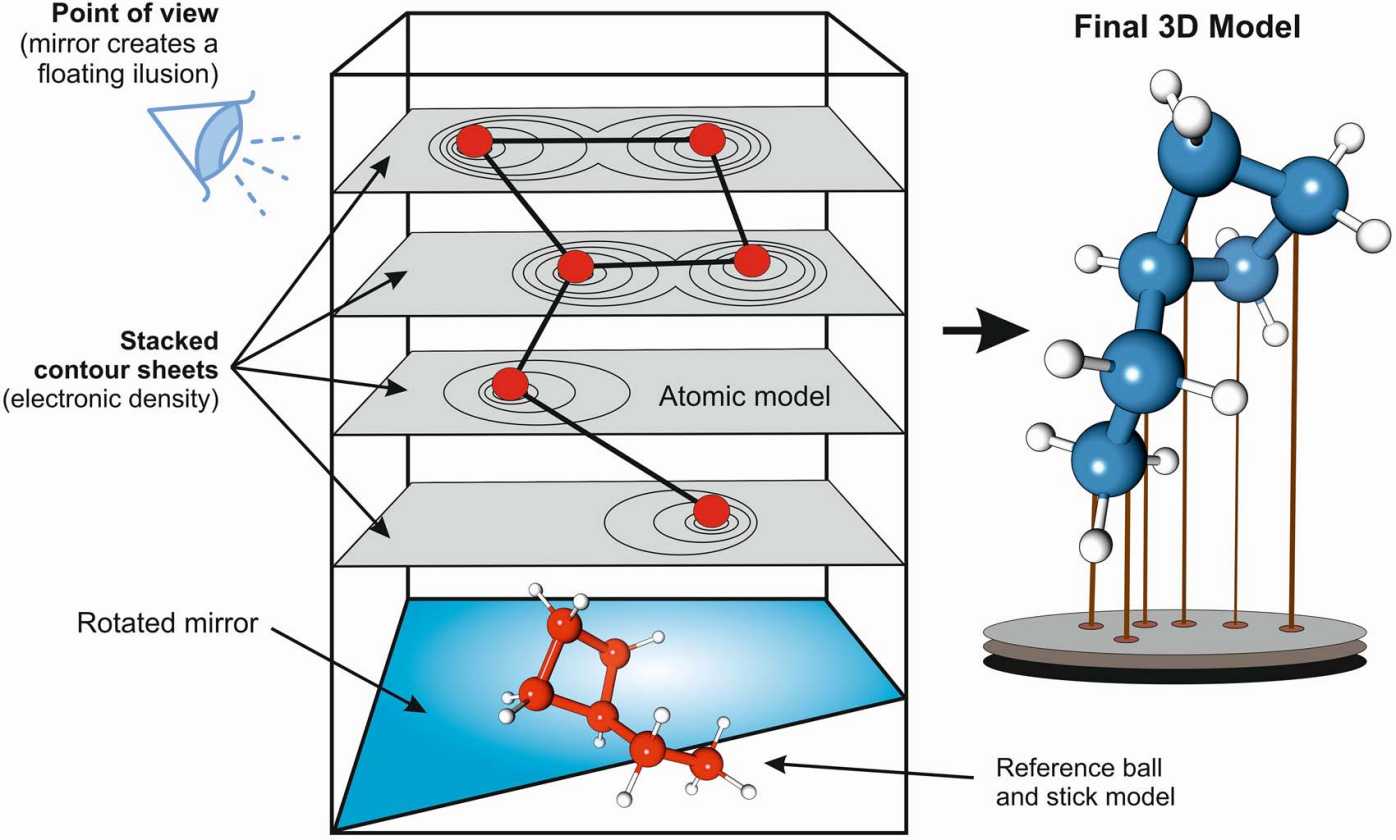
Schematic representation of the Richards Box or “Freds Folly,” an early optical device used to interpret electron density maps in protein crystallography. The system consisted of stacked transparent sheets displaying contour lines of electron density, aligned in three dimensions, and separated by regular spaced intervals. A tilted semitransparent mirror at the base projected the image of a reference ball and stick model to create the illusion of a floating molecule when observed from a fixed point of view. Atomic positions in the reference structure could be modeled and refined within the electron density contours, enabling the construction of the preliminary molecular model. The resulting interpretation was subsequently translated into a physical ball-and-stick model, serving as a reference for structural analysis.

In 1968, Fred M. Richards developed the “optical comparator,” later known as the Richards Box and nicknamed as “Fred’s Folly,” to physically superimpose 3D electron density with an atomic model at a scale of ~1 cm per Å [7]. The apparatus used contour lines from Fourier-calculated electron-density maps, which were manually traced onto a series of transparent plexiglass sheets, each corresponding to a parallel section at defined Z-axis intervals (Fig. 2). A semitransparent mirror, positioned at roughly 45°, optically merged the illuminated stack of contour plates with a Kendrew-style skeletal model of wires and brass rods mounted perpendicularly below. This optical arrangement produced the illusion of the atomic model “floating” within the electron density, allowing the crystallographer to perceive both representations. By bending wires or repositioning atoms to align the skeletal model with the iso-contours, researchers could iteratively build and refine atomic coordinates with direct 3D feedback, an achievement that preceded the advent of interactive computer graphics by several years. Later adaptations included smaller or modified versions constructed by contemporary laboratories, while the underlying concept inspired early “electronic Richards boxes” and eventually evolved into computer-assisted model-building programs such as FRODO [8] and O [9].

Operationally, the Richards box offered key advantages. Its optical superposition system ensured one-to-one correspondence between the physical model and the electron-density map, providing accurate spatial registration in depth. The device was inexpensive, simple to assemble, and it enabled intuitive model building by preserving continuous 3D context across stacked sections. However, it also had clear limitations: model construction was labor-intensive, dependent on manual plotting and contour alignment, and prone to parallax and optical distortion. Its bulk, limited Z-axis sampling set by plate spacing, and the difficulty of updating maps as phase information improved, further constrained its utility. By the late 1970s, this manual optical approach was largely replaced by interactive molecular graphics systems, which retained the core task of fitting atomistic models to density while eliminating mechanical and optical constraints. Even so, the Richards box played a key transitional role in standardizing model-building practices and legitimizing protein crystallography during a period when few macromolecular structures were known.

The limitations of physical molecular models became clear as protein structures grew in complexity beyond what manual assembly could represent. Although useful for verifying atomic positions and major folding features, these models were inefficient for recognizing secondary-structure elements such as α-helices, β-sheets, or overall chain topology. Dense atomic detail often obscured higher-order organization, underscoring the need for new representational strategies that could distill atomic information into more informative graphical abstractions. This conceptual shift led to the development of schematic ribbon diagrams, introduced by Jane Richardson in the 1980s [10], and to the emergence of computer-assisted molecular graphics systems that replaced static physical models with dynamic, scalable, and more interpretable visualizations [11]. The pioneering structural determinations by Perutz and Kendrew thus not only provided the first detailed views of protein architecture but also revealed the inherent limitations of physical models, driving the transition of molecular representation into the computational era of structural biology.

## Cyrus Levinthal and the “Kluge”

Cyrus Levinthal’s group at MIT built what is widely regarded as the first interactive molecular graphics system, coupling a time-shared mainframe on Project MAC to a custom, oscilloscope-like vector display terminal nicknamed the “Kluge”. The display rendered wireframe models of proteins by storing 3-D atom–bond vectors and continuously projecting them to the screen while the model rotated, which provided depth perception without stereoscopy. A dome-shaped “globe” input (a forerunner of the more recent trackball devices) allowed the user to control rotation speed and direction in real time; additional routines let users manipulate torsion angles and polypeptide chains, enabling model-building experiments on segments of proteins such as myoglobin and lysozyme [12]. The system also embodied early computational strategies to keep interaction responsive on limited hardware, for example, “cubing” space and checking short-range interactions only against atoms in the same or adjacent cubes (a divide-and-conquer neighbor-list idea). Beyond the live console work, the group produced 16-mm films of animated protein motion from sequences of display frames, making the results portable to labs without access to the terminal. The University of Massachusetts still maintains a website containing pictures of the original Levinthal’s hardware and some early animations generated by the system (https://www.umass.edu/molvis/francoeur/levinthal/lev-index.html).

Levinthal’s design delivered two decisive advantages over physical CPK models (a color-coding convention used for molecular models, named after its creators Corey, Pauling, and Koltun, that assigns specific colors to atoms to distinguish elements in 3D, space-filling models, e.g. Carbon = Black, Hydrogen = White, Oxygen = Red, Nitrogen = Blue) and batch plotting: true interactivity with immediate, continuous visual feedback while exploring conformational changes and steric contacts; and computational filtering of local interactions that helped scientists focus on chemically plausible arrangements [12]. In 1966, Cyrus Levinthal framed the system explicitly as a “man–computer combination” arguing that human chemical insight coupled to computer graphics could guide searches through conformational space more effectively than unaided computation or static models [13]. However, the system suffered from some limitations. The Kluge was monochrome, line-drawn, and low-resolution; in consequence, there was no hidden-line removal or shaded surfaces, so depth cues depended on perpetual rotation of the models. Memory and Central Processing Unit (CPU) constraints conditioned the model size and forced algorithmic simplifications; much of the energy evaluation remained approximate, and Levinthal later emphasized that the full prediction of protein folds interactively had been “grossly over-optimistic” at the time [14]. The terminal was unique and fragile, making the setup difficult to reproduce elsewhere until newer displays and minicomputers appeared in the late 1960s and early 1970s. Nevertheless, the system established the core paradigm of interactive molecular graphics, real-time rotation/selection, torsion manipulation, neighbor-limited contact checks, and cinematic output, that directly influenced subsequent molecular graphics platforms [15].

From an algorithmic perspective, the “cubing” strategy employed by Levinthal’s system is an early instance of the cell-list (spatial hashing) algorithm, which partitions 3D space into a regular grid and restricts pairwise interaction checks to atoms within the same or adjacent cells. This reduces the computational complexity of neighbor searches from O(N^2^) to O(N) [16], a reduction that remains the basis of contact detection in all modern molecular dynamics engines and real-time visualization systems. The fact that Levinthal’s group implemented this strategy in the mid-1960s, on hardware with kilobytes of memory, underscores the algorithmic ingenuity that underpinned the earliest interactive molecular graphics.

## Evans & Sutherland computers: the first generation of computer molecular graphics

In 1968, the Evans & Sutherland company (E&S) was established in Salt Lake City by David C. Evans and Ivan Sutherland as a spin-off initiative from the University of Utah, with the goal of commercializing interactive computer graphics. The company’s first major product, the Line Drawing System-1 (LDS-1, 1969), was a calligraphic vector display processor tightly integrated with host computers such as DEC systems. It implemented 4 × 4 transformation pipelines (a sequence of mathematical operations used in 3D computer graphics to convert object coordinates into screen-space pixels that relies on 4 × 4 matrices to perform linear transformations—such as rotation, scaling, translation, and perspective projection—using homogeneous coordinates) capable of animating complex wireframe models in real time, features that positioned the LDS-1 as a prototype for modern GPUs. Building on this success, E&S introduced a series of “Picture System” vector workstations (PS1/PS2 and the PS300 family) and, in 1973, unveiled the Shaded Picture System, the first commercial platform capable of real-time hidden-line removal and shaded 3D rendering, albeit with limited raster resolution. These pioneering systems, along with later E&S display technologies, formed the foundation for 3 decades of advancements in visual simulation, computer-aided design, and scientific visualization, until raster-based UNIX workstations ultimately supplanted vector displays in the late 1980s and 1990s.

In macromolecular crystallography and molecular modeling, Evans & Sutherland (E&S) systems became established as the predominant high-end platforms for interactive 3D visualization from the late 1970s to the mid-1980s. The transition from optical model-building approaches, exemplified by the Richards Box, to computer-based graphics was driven by Alwyn Jones, who implemented his program FRODO on an E&S Picture System 2 interfaced with a PDP-11 [9]. This configuration enabled real-time rotation, zooming, and coordinate editing directly within contoured electron-density maps, revolutionizing the model-fitting process. Similar adaptations and successor programs—such as the University of North Carolina’s GRINCH and later molecular docking interfaces—took advantage of the PS-series’ smooth, low-latency vector rendering and depth-cueing capabilities, which reduced operator fatigue and accelerated structural interpretation [8]. Contemporary reports indicate that, despite their high cost (approximately USD 250 000 in 1985), E&S systems were widely adopted in crystallographic laboratories. Nonetheless, these platforms exhibited significant limitations, including constraints on memory and geometric complexity inherent to vector lists, the inflexibility of the PS300’s dataflow display architecture for computational tasks such as map calculation or energy evaluation, and the absence of high-quality shaded raster output until later generations. These factors ultimately drove the community’s migration toward raster-based UNIX workstations and, subsequently, to commodity GPUs and modern tools such as O [17] and Coot [18].

Taken together, the Evans & Sutherland LDS-1 and Picture System series constituted the first widely adopted commercial infrastructure for interactive 3D scientific visualization. In the context of molecular graphics, these systems marked the transition from hand-drawn and optical compositing techniques to software-based, real-time model building and analysis. They established fundamental interaction paradigms, such as continuous 3D manipulation, depth cueing, stereoscopic visualization, and responsive model editing (understood as the possibility of introducing structural changes in the depicted model in a dynamic way), that remain integral to modern molecular graphics environments. The LDS-1’s 4 × 4 transformation pipeline implements homogeneous coordinate transformations, a mathematical framework in which rotation, translation, scaling, and perspective projection are unified into a single matrix multiplication. This representation became the universal basis of all subsequent graphics hardware and is directly embodied in the vertex shader stage of modern GPUs. The 1973 Shaded Picture System introduced real-time hidden-surface removal using a z-buffer (depth buffer) approach [19], which stores the depth of the nearest rendered fragment at each pixel and discards occluded geometry in O(1) per fragment—a dramatic improvement over the O(N log N) painter’s algorithm it replaced. These two algorithmic contributions—homogeneous matrix pipelines and z-buffering—are not merely historical curiosities; they are the direct ancestors of the rendering pipelines embedded in every modern GPU and molecular graphics application.

## Kinetic images and Rasmol: democratization of molecular visualization

In the early 1990s, David and Jane Richardson introduced the kinetic images or “kinemages.” The kinemage format is a lightweight, plain-text medium for interactive 3D scientific figures. Conceived during the transition from specialized graphics hardware to general-purpose UNIX workstations, the format embodied the growing accessibility of molecular visualization. Rather than functioning as a general-purpose molecular model file, a kinemage served as an author-curated interactive illustration, designed to communicate specific structural features such as fold motifs, active-site geometries, or packing interactions. Users could freely rotate models, toggle components, identify points, measure distances, and animate transitions between related conformations with near-instant responsiveness. Technically, a kinemage encoded hierarchical groups, lists, and points constructed from simple graphical primitives (points, vectors, and ribbons) supplemented by color definitions and toggle controls. The format was both human- and machine-readable, platform-independent, and easily archived. Authors should employ two different pieces of software to generate and manipulate kinemages: PREKIN to convert Protein Data Bank (PDB) coordinates into the kinemage format and MAGE to view or edit them. Beginning in 1992, “Protein Science” distributed these interactive figures alongside research articles in a floppy disk, establishing the kinemage as one of the first digital supplements in structural biology and a pivotal step toward the fully interactive molecular graphics environments that followed [20].

**Figure 3:**
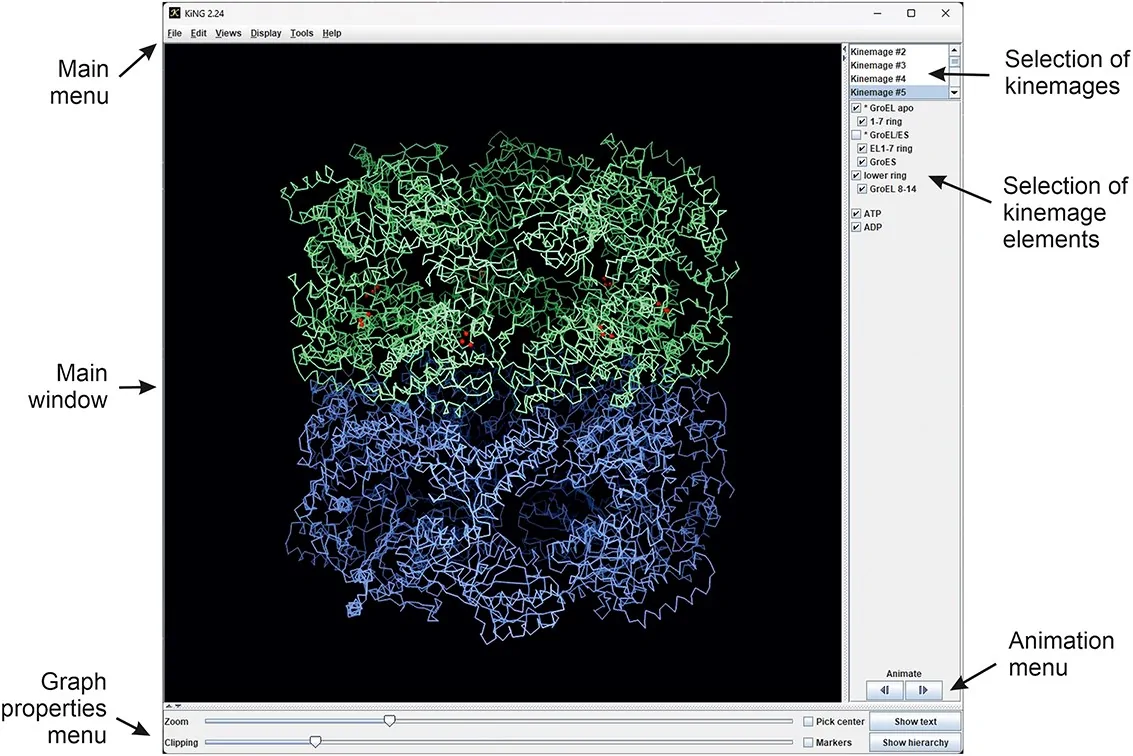
Graphical interface of the *KiNG* (Kinemage, Next Generation) molecular visualization software. The main window displays a structural representation of the GroEL–GroES complex as a kinemage model as an example of visualization, with the upper and lower rings shown in distinct colors. The top bar provides access to the main menu drop sections (file, edit, views, display, tools, help), while the lower section includes the graph properties menu, allowing control of zoom, clipping, and display options. On the right panel, users can select among multiple loaded kinemages and toggle visibility of individual structural elements such as subunits, ligands, or annotations. The animation menu enables playback of predefined motions or conformational transitions encoded within the kinemage file. This interface exemplifies the interactive, hierarchical design of KiNG, facilitating user-guided exploration and annotation of 3D macromolecular structures.

The philosophy behind the format emphasized clarity, pedagogy, and accessibility: authors guided readers through the essential “signal within structural complexity,” while maintaining interaction and reproducibility through simple ASCII text files [21]. The Kinemage format is a plain-text, hierarchical description of interactive 3D vector graphics, originally developed for molecular visualization, in which a scene is defined through nested elements, like headers, groups, subgroups, and lists, that organize graphical primitives such as points, vectors, balls, and ribbons, each associated with 3D coordinates and semantic labels (e.g. atoms, residues, or interactions). Its structure is both human-readable and modular, allowing users to encode not only geometry but also biological meaning and display logic. Customization is achieved by modifying or extending these elements: users can control visibility through group and master toggles, define colors and styles (e.g. line width, sphere radius), create multiple predefined views (camera orientation, zoom, center), and annotate features with labels or text blocks. Because it is text-based, Kinemage files can be easily edited manually or generated programmatically, enabling flexible adaptation for teaching, structural analysis, or highlighting specific molecular features such as domains, binding sites, or dynamic interactions. This design ensured longevity, enabling files to be compared, versioned, or generated by scripts. Kinemages offered major advantages. In teaching, they provided intuitive tools for exploring macromolecular structure, hydrogen bonding, topology, or packing, without requiring advanced graphics hardware. In research, the MAGE/PREKIN system used kinemages for live visualization of all-atom contacts and conformational analysis, integrating visualization with validation workflows. The later KiNG viewer (Kinemage Next Generation viewer), a Java-based successor, preserved compatibility while adding plugins for advanced data visualization, such as rotamer plots and structure rebuilding tools (Fig. 3) [22].

Kinemages also became important tools for validation pipelines like MolProbity [23], enabling interactive inspection of Ramachandran plots, rotamer distributions, and contact outliers. Their adaptability extended into immersive visualization such as KinImmerse system for VR in NMR ensembles, illustrating the enduring flexibility of the text-based representation [24]. Historically, the kinemage ecosystem, comprising PREKIN, MAGE, and later KiNG, democratized interactive molecular graphics by providing accessible, interpretable 3D tools long before web-based viewers existed (Fig. 3). By prioritizing clarity and interactivity over photorealism, kinemages set enduring standards for transparent, reproducible, and pedagogically effective visualization in structural biology. For further resources, see the original project website at the Richardsons’ laboratory (http://kinemage.biochem.duke.edu).

In parallel with the kinemage initiative, RasMol emerged as one of the first widely accessible ANSI C-based viewers for macromolecular coordinates. Its development began in the early 1990s, with the software first described in 1992 and followed shortly thereafter by the initial public releases of the 2.x series. Designed for speed, portability, and simplicity, RasMol ran efficiently on personal computers across multiple operating systems, democratizing molecular visualization beyond the domain of specialized graphics workstations [25]. Its design priorities, portability, interactive speed on modest hardware, and a compact command language, made publication-quality representations accessible to labs and classrooms worldwide. Subsequent stewardship and consolidation of variants by Herbert J. Bernstein led to the “Open RasMol” stream (2.7 series), with licensing evolving through RASLIC to GPL for current distributions [26]. Conceptually, RasMol differed from the Richardson’s kinemage ecosystem. Kinemages are author-designed, interactive vector graphics accompanying publications, intended to convey specific 3D concepts, whereas RasMol is a versatile, scriptable molecular viewer that directly reads coordinate and map files, enabling users to construct customized visualizations, selections, and measurements in real time. In short, kinemages prioritize communication of predefined insights and RasMol prioritizes exploratory visualization on commodity machines. Both were responsible for the democratization of molecular graphics in the early 1990s.

## Integrated environments for macromolecular visualization and analysis: a comparative overview of PyMOL, ChimeraX, and YASARA

Modern structural biology relies on interactive software that unifies high-quality 3D visualization with tools for analysis, modeling, and communication. Among widely used platforms, PyMOL [27], UCSF ChimeraX [28], and YASARA [29] exemplify different design philosophies: PyMOL emphasizes publication-grade rendering and a rich plugin ecosystem; ChimeraX targets next-generation data types (e.g. large cryo-EM maps and light-microscopy volumes) with GPU-accelerated graphics and modern interfaces; and YASARA integrates visualization tightly with built-in molecular modeling and dynamics.

**Figure 4:**
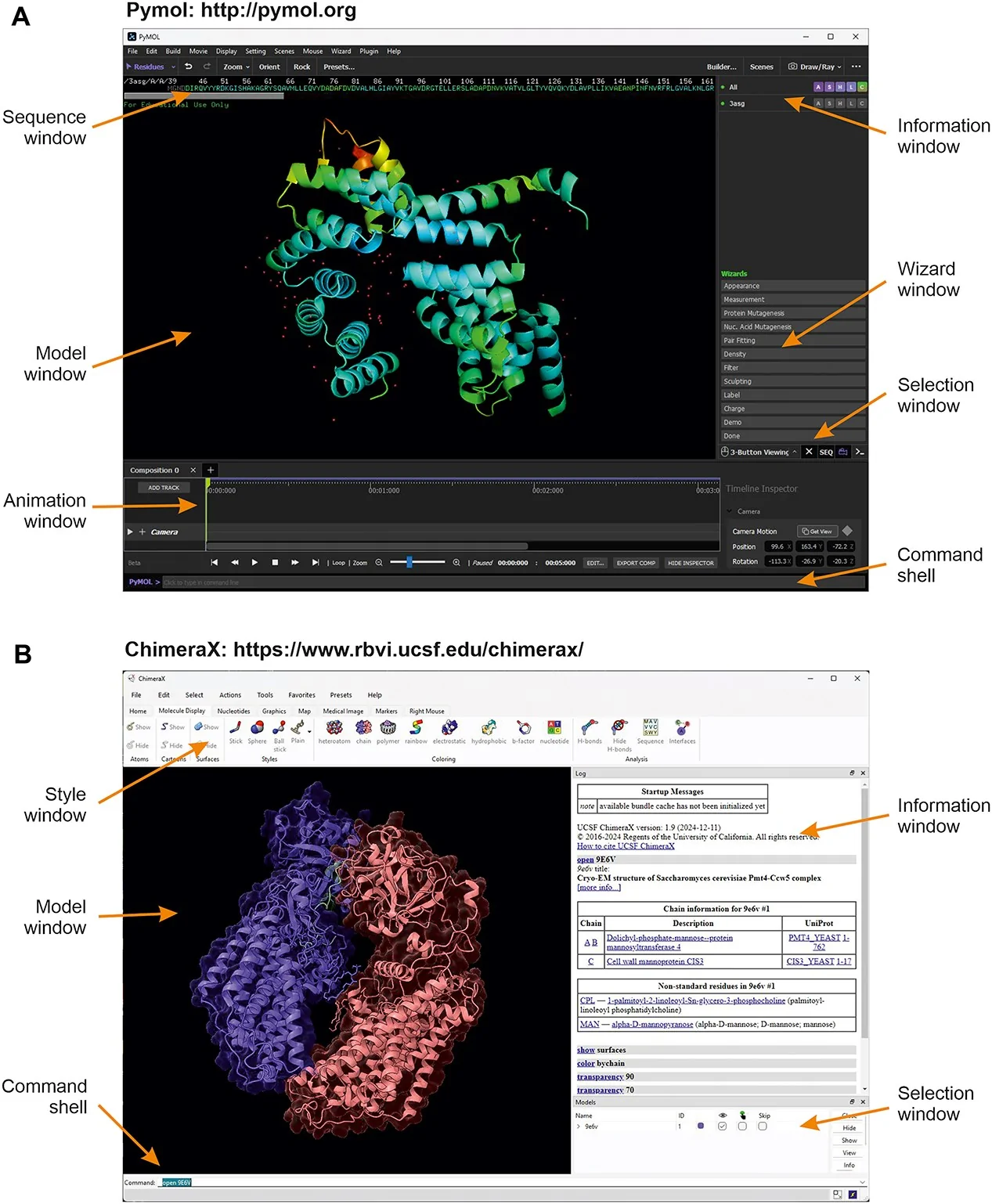
Graphical interfaces of molecular visualization environments PyMOL and UCSF ChimeraX. (A) Screenshot of the PyMOL interface showing the main interactive components: sequence, model, and animation windows, complemented by information, wizard, selection, and command panels. PyMOL provides a script-based yet user-friendly environment for molecular modeling, visualization, and animation. (B) Screenshot of the UCSF ChimeraX interface illustrating the integrated model display, style control, and command shell, together with information and selection windows. ChimeraX combines modern graphics capabilities with efficient molecular rendering and advanced structural analysis tools.

PyMOL was conceived and developed in the late 1990s by Warren Lyford DeLano as an open-source molecular graphics system designed to make high-quality visualization and scripting accessible to the structural biology community [27]. Initially released in 2000 under the Python-based DeLano Scientific distribution, PyMOL introduced an innovative combination of a powerful command-line interface, an extendable Python API, and advanced 3D rendering capabilities that allowed scientists to create publication-quality molecular figures and animations with unprecedented control (Fig. 4A). PyMOL’s high-quality ray-traced images employ ambient occlusion, a screen-space approximation of global illumination that estimates the fraction of the hemisphere above each surface point that is occluded by nearby geometry. This technique, adapted from real-time rendering, dramatically improves depth perception in molecular images without the computational cost of full ray-tracing. DeLano’s guiding philosophy, open-source, open access, and open science, fostered a vibrant user and developer community that quickly expanded PyMOL’s capabilities through plugins and custom scripts [30, 31]. Following DeLano’s untimely death in 2009, maintenance of the project was assumed by Schrödinger, Inc**.**, which continues to develop the commercial version while maintaining a community-supported open codebase [32]. Over the years, PyMOL has become one of the most influential tools in molecular visualization, widely adopted in research, education, and pharmaceutical design, owing to its balance between visual esthetics, scripting flexibility, and scientific rigor. PyMOL delivers fast, precise molecular graphics with fine control over representation, lighting, ray-tracing, and figure composition. Its feature set covers structural superposition, density map display, simple morphing/animations, measurement tools, and movie production, making it a default choice for figure-centric workflows and teaching. The current PyMOL distribution, maintained by Schrödinger, builds upon its original open-source foundation but functions largely as a commercial platform, with regular releases, technical support, and extended feature sets. PyMOL provides a mature Python API and a robust, long-standing plugin ecosystem, including modules for surface calculations [33], molecular docking [34], and trajectory [35] or contact-map analysis [36]. Plugin development using PyQt is well documented, and curated repositories facilitate the discovery and integration of community-contributed add-ons, sustaining PyMOL’s relevance as a flexible and extensible environment for structural biology [37]. For atomic models and high-end static figures, PyMOL’s ray-tracer and artistic controls (ambient occlusion, depth cueing, shadows) remain a benchmark, which also includes a group of robust movie tools for communication and teaching. Limitations become apparent when handling exceptionally large datasets or performing tasks that demand deep integration with volumetric image analysis, areas in which other software such as ChimeraX demonstrates superior performance.

UCSF ChimeraX [28] is the successor to UCSF Chimera [38], redesigned for modern GPUs and large datasets. The performance gains over its predecessor are substantial: ChimeraX has been benchmarked against whole-cell models containing over 114 million particles, a scale entirely inaccessible to earlier CPU-based viewers [39]. It provides interactive visualization of atomic models, cryo-EM maps, 3D light-microscopy volumes, and associated annotations, along with analysis tools for fitting, segmenting, morphing, and comparative modeling workflows (Fig. 4B). Notably, ChimeraX includes VR interaction options and specialized tools for handling very large density maps. It is free for non-commercial use and extensively documented in peer-reviewed articles and working software manuals [28]. ChimeraX offers both a concise command language and Python scripting, with many tasks (selection logic, morphing, map filtering, model building) scriptable for reproducibility. Its functionality is delivered through the core package and RBVI-supported channels (Resource for Biocomputing, Visualization, and Informatics, from University of California San Francisco, UCSF) rather than an extensive third-party plugin ecosystem, which streamlines maintenance but may restrict specialized extensions compared to PyMOL. ChimeraX employs level-of-detail (LOD) rendering strategies, dynamically reducing polygon counts for distant or less-critical regions of large assemblies, enabling interactive visualization of viral capsids containing millions of atoms at frame rates exceeding 30 fps on standard workstation hardware [40]. Peer-reviewed papers describe the command architecture and session reproducibility features. ChimeraX was engineered for responsiveness on large cryo-EM maps and multiscale scenes, leveraging GPU-accelerated volume rendering, LOD strategies, and VR interaction. It supports interactive map filtering, fitting, and segmentation alongside atomic models, easing end-to-end cryo-EM interpretation and presentation [41]. ChimeraX provides rich comparative analysis, dynamic transitions, and graphical interpolation between molecular states (morphing), distance/angle networks, interface analysis, and connections to modeling resources. It handles hybrid pipelines—atomic models plus EM maps or microscopy volumes but does not position itself as an all-in-one MD engine. ChimeraX is free for non-commercial use with installers for Windows, macOS, and Linux, and is actively developed by RBVI with extensive user documentation and an active release cadence documented in the literature [42].

YASARA (Yet Another Scientific Artificial Reality Application) is a versatile molecular modeling and visualization software that integrates a broad range of computational tools within a single, interactive environment [29]. It supports molecular graphics, structure building, energy minimization, molecular dynamics (MD) simulations, and docking, offering a continuum between visualization and simulation. Its rendering engine provides high-quality graphics with real-time manipulation, allowing users to visualize macromolecular structures, electrostatic surfaces, and dynamic trajectories efficiently, even on modest hardware [29]. The scale of this challenge is quantitative: rendering a single atom as a convincing sphere requires ~320 triangles, so a 10 000-atom protein demands over 3 million triangles per frame—far exceeding standard GPU rasterization throughput [29]. YASARA’s hybrid CPU–GPU algorithm circumvents this bottleneck by computing perfectly round spheres without triangle tessellation. At the upper extreme of scale, YASARA’s Vulkan-based engine has been used to build and interactively visualize an all-atom model of an entire presynaptic bouton containing 3.6 billion atoms [43]. YASARA is particularly appreciated for its user-friendly interface and integrated Python-based macros, which facilitate automation and reproducibility of complex workflows. Unlike many visualization programs that rely on external packages, YASARA includes built-in force fields and solvation models, making it well suited for rapid prototyping and educational use [44]. However, some limitations persist: the free version restricts access to advanced modeling functions, and the software’s proprietary nature limits community-driven development and plugin expansion compared to fully open-source alternatives like PyMOL or ChimeraX. Additionally, its graphics, while performant, can be less customizable than those in specialized rendering tools. Overall, YASARA offers an effective balance between accessibility and functionality, serving both as an introductory platform for molecular modeling and as a competent environment for small- to medium-scale simulations and visualization tasks [45].

## The Collaborative Computational Project Number 4 (CCP4) and its visualization software: CCP4mg and Coot

The Collaborative Computational Project Number 4 (CCP4) was founded in 1979 under the auspices of the UK Science and Engineering Research Council, with the mission of supporting protein crystallography through the distribution of a unified suite of software for data processing, phasing, model building, refinement, and validation [46]. Before CCP4’s establishment, crystallographic computing relied on heterogeneous, often institution-specific codes, creating compatibility and reproducibility challenges. By integrating algorithms and data formats under a shared framework, CCP4 revolutionized structural biology, becoming the reference platform for macromolecular crystallography [47]. As crystallographic datasets grew in size and resolution, the need for sophisticated visualization and model-building tools capable of interfacing directly with CCP4 outputs led to the development of Coot and CCP4mg, two of the most influential molecular graphics applications derived from this project [48].

Coot (Crystallographic Object-Oriented Toolkit), initiated by Paul Emsley in the early 2000s, filled a crucial gap in the crystallographic workflow: real-time, interactive model building within electron-density maps [18]. Before Coot, researchers often relied on non-specialized molecular graphics programs such as O or XtalView [49], which required manual synchronization between density maps and coordinate data. Coot introduced a new paradigm by coupling graphical interactivity with crystallographic computation, allowing users to visualize, manipulate, and refine macromolecular models in three dimensions while maintaining strict stereochemical restraints [18]. Its underlying architecture—written in C and Python—enabled both performance and extensibility, while the adoption of the CCP4 map and MTZ file standards ensured seamless integration with established refinement engines such as REFMAC5 [50], PHENIX [51], or BUSTER [52].

**Figure 5:**
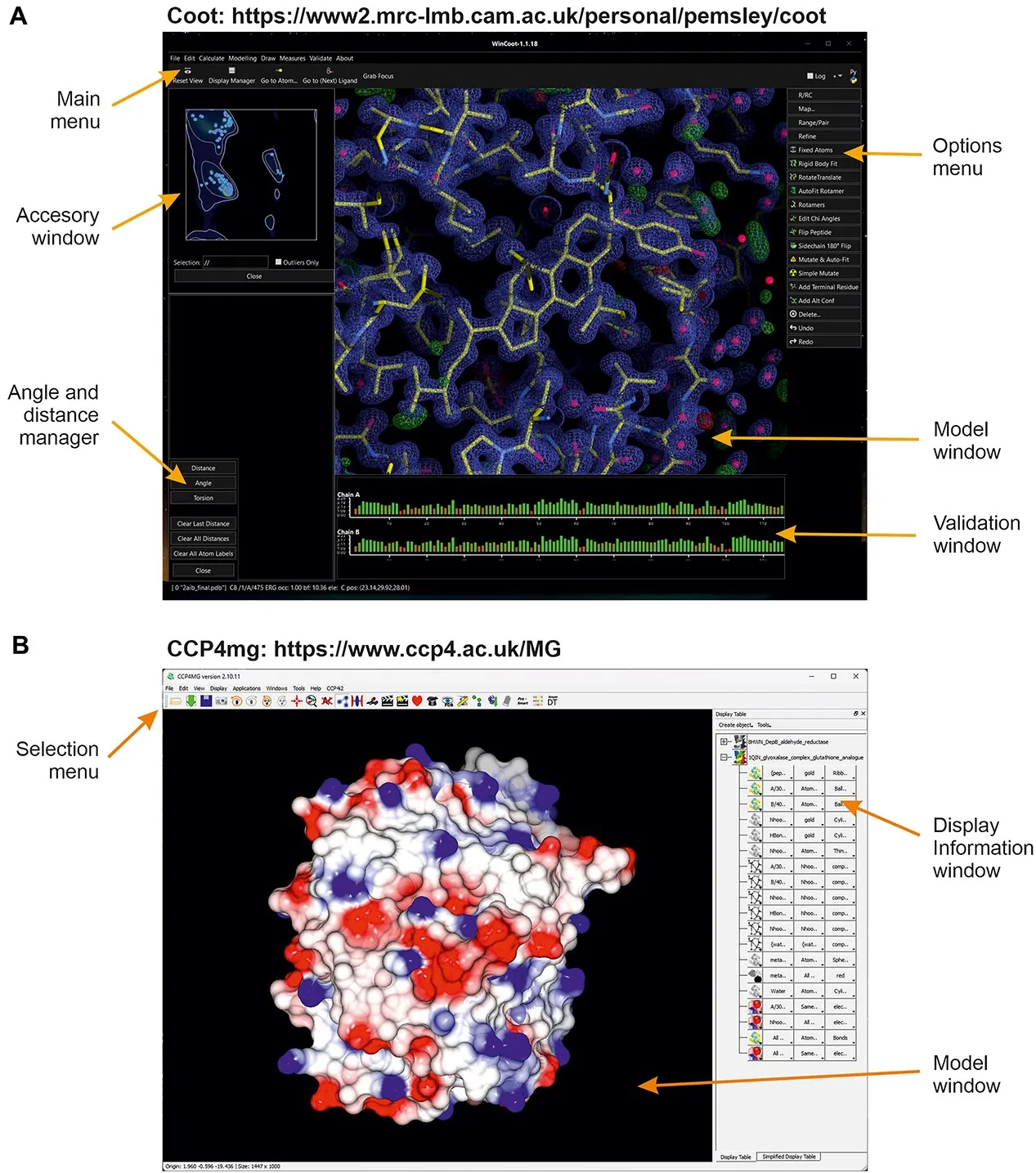
Visualization interfaces of CCP4-related molecular graphics software. (A) Coot (crystallographic object-oriented toolkit) provides an interactive environment for model building and electron-density fitting in macromolecular crystallography. The screenshot illustrates the main interface components, including the main and accessory windows, the angle and distance manager, the options and validation panels, and the central model window displaying the fitted atomic model within the electron-density map. (B) CCP4mg (CCP4 molecular graphics) is a versatile molecular visualization program integrated within the CCP4 suite. The image shows the user interface highlighting the selection menu, display information window, and the model window depicting an electrostatics surface representation. Both tools exemplify the visualization and model refinement capabilities developed within the Collaborative Computational Project No. 4 (CCP4) framework for structural biology.

From a functional perspective, Coot provides a powerful set of tools for map fitting, ligand placement, model validation, and local real-space refinement [53]. Features such as dynamic difference-map updating, automated ligand fitting, and real-time validation plots (Ramachandran, rotamer, and geometry checks) made it the preferred software for crystallographers seeking immediate visual feedback during model building (Fig. 5A) [54]. The user interface, while originally minimalist, evolved to include scripting capabilities and plugin extensions, facilitating automation and reproducibility [55]. One of Coot’s key strengths is its tight integration with the CCP4 environment, enabling direct inspection of refinement statistics, electron-density quality, and model geometry within a single environment [56]. Despite these advantages, Coot’s reliance on local computational resources and its steep learning curve for beginners are often cited as limitations. Moreover, its rendering engine, though efficient for crystallographic purposes, is less suited for producing photorealistic visualizations compared to modern GPU-based systems such as ChimeraX [41] or PyMOL [57]. The interactive display of electron-density maps in Coot relies on the Marching Cubes algorithm [58], which extracts a polygonal isosurface from a 3D scalar field in O(N) time by processing each voxel independently. Originally published at SIGGRAPH 1987 and now among the most cited papers in computer graphics (>15 000 citations), this algorithm transformed the display of crystallographic maps from static contour plots into dynamically adjustable, real-time 3D surfaces. The ability to interactively adjust the contour level and immediately observe the resulting surface—a workflow central to model building in Coot—is a direct consequence of the O(N) complexity of the Marching Cubes approach.

In parallel, the CCP4 team developed CCP4mg (CCP4 Molecular Graphics) as a more general-purpose visualization program, emphasizing molecular analysis, figure preparation, and educational usability rather than model editing. Initially conceived in the early 2000s by Stuart McNicholas, Eleanor Potterton, and colleagues at the CCP4 group, CCP4mg sought to provide a versatile viewer capable of interpreting both atomic and volumetric data [59]. Built on the CCP4 software libraries, CCP4mg supports a broad range of file formats (PDB, mmCIF, MRC, CCP4, and EM maps) and includes functionalities for displaying electron densities, electrostatic surfaces, secondary-structure cartoons, and molecular assemblies (Fig. 5B). Importantly, it introduced a high-quality rendering system that allowed users to prepare publication-ready figures directly from refined coordinates, including shadows, transparency, and molecular surfaces colored by properties such as electrostatic potential or B-factor [60].

CCP4mg’s integration with the CCP4 suite was not merely technical but conceptual—it reflected CCP4’s philosophy of reproducible and open science. The program allows the automatic loading of experimental maps and refined models from CCP4 project directories, reducing the need for manual configuration [61]. Its scene scripting feature provides a robust mechanism for generating reproducible visualizations and animations, which can be stored and replayed across sessions. These characteristics made CCP4mg particularly valuable in teaching environments and collaborative projects, where consistency of graphical output is critical. On the downside, CCP4mg has faced criticism for limited interactivity and slower performance when handling large assemblies, particularly compared to GPU-accelerated molecular viewers. The pace of its development has also slowed in recent years, though it remains maintained within the CCP4 distribution as a stable and reliable visualization tool [60].

Together, Coot and CCP4mg embody the long-standing vision of the CCP4 initiative—to provide crystallographers with a cohesive, open, and scientifically rigorous ecosystem for structural determination. Coot represents the experimentalist’s workbench, enabling precise model construction and validation within electron-density maps, while CCP4mg serves as the communication interface, transforming structural data into comprehensible and visually appealing representations. Their historical development highlights the transition in macromolecular crystallography from command-line-driven computation to interactive, visualization-oriented science, bridging computation and human perception. Despite competition from newer visualization platforms, the enduring use of Coot and CCP4mg underscores their robustness, accessibility, and deep integration into crystallographic workflows. Both remain widely cited and continuously used in structural biology laboratories worldwide, a testament to CCP4’s sustained impact on the discipline.

## Web-based platforms for molecular graphics

The recent evolution of web technologies has enabled the emergence of powerful, browser-based molecular visualization environments capable of handling increasingly complex structural data. Together, these web-based visualization frameworks represent a major shift from standalone software to cloud-integrated, platform-independent molecular analysis, broadening accessibility and supporting collaborative, data-driven research.

**Figure 6:**
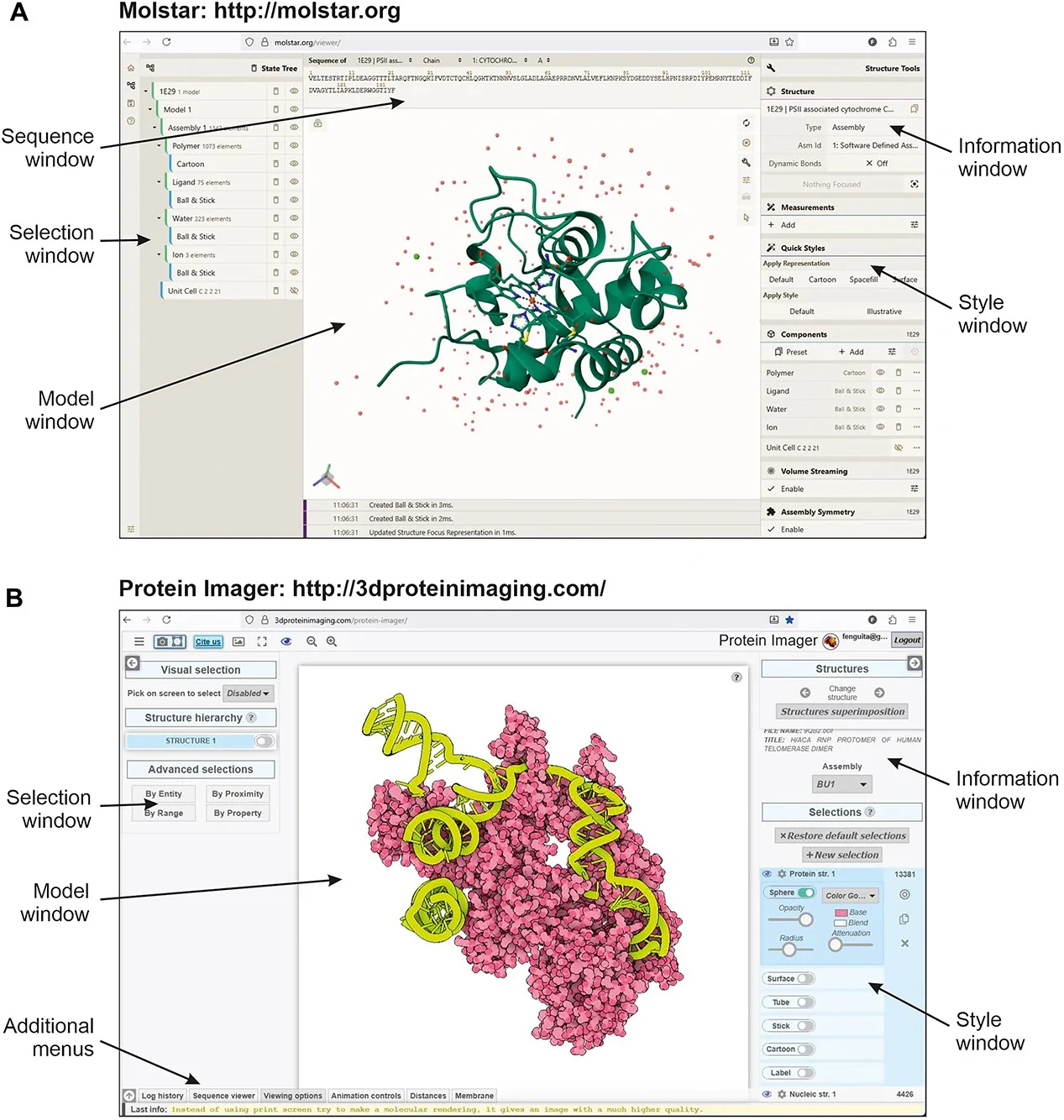
Web-based molecular visualization interfaces of Mol (MolStar) and Protein Imager. (A) Screenshot of MolStar (available at http://molstar.org) illustrating its modular interface comprising a sequence window, a selection and model hierarchy panel, and a central model window for interactive molecular visualization. The right panel contains information and style control windows that allow users to adjust molecular representations, color schemes, and display parameters in real time. (B) Screenshot of Protein Imager (available at http://3dproteinimaging.com) showing its structured layout with selection and advanced selection panels on the left, a model window for 3D rendering of biomolecular structures, and information and style windows on the right for modifying display options. Additional menus at the bottom provide access to animation, membrane, and distance analysis tools. Both applications enable interactive, browser-based exploration of macromolecular structures without the need for local installation.

Mol* (often referred to as MolStar) is a modern web-native molecular graphics engine developed jointly by PDBe and RCSB PDB teams, merging features from previous viewers such as LiteMol [62] and NGL [63]. Nowadays MolStar is the backbone of 3D visualization in major structural biology portals, including the PDB database [64]. The scale of structures that MolStar can handle is quantitatively unprecedented for a browser-based tool: the system supports structures containing hundreds of millions of non-hydrogen atoms, including integrative models of entire macromolecular machines [64]. Its predecessor LiteMol demonstrated that the HIV capsid—comprising 2.4 million atoms—could be loaded and rendered interactively in a web browser in under 1 minute [62]. This performance relies in part on the Macromolecular Transmission Format (MMTF), a binary compressed format that achieves over 75% size reduction compared to standard mmCIF files and is more than 10 times faster to parse, enabling interactive visualization of million-atom complexes on smartphones as well as desktop computers [65]. MolStar supports streaming of coordinate and volumetric data (e.g. PDB/mmCIF, EMDB maps) and can handle very large biomolecular assemblies, including integrative/hybrid models and cell-scale environments with millions of atoms. It offers interactive tools for selection, measurement (distances/angles/dihedrals), model superposition, symmetry exploration, density map slicing and isocontour display, and annotations or overlays such as B-factor coloring, ligand interactions, or validation metrics (Fig. 6A). Through modular design, MolStar can also integrate extensions such as Molecular Volumes and Segmentations (Mol VS), which provides specialized handling of volumetric and segmentation data (e.g. segmented maps, annotation editing, multi-frame channels) [64]. As a full-featured viewer, MolStar offers a mature user interface with hierarchical scene controls, command logging, presets, and scripting hooks. Since it is open source and web-based, it integrates directly into data portals and custom web services. Its architecture (TypeScript, WebGL, React) ensures modern web performance and maintainability. However, new users may face a steep learning curve due to the richness of its features and the depth of options. Moreover, rendering large scenes may challenge less powerful client hardware or mobile devices, potentially leading to lag or dropped frames.

The transition to browser-based molecular visualization was enabled by the standardization of WebGL (2011), which exposes the GPU’s programmable shader pipeline to JavaScript applications. Unlike earlier software renderers, WebGL allows molecular geometry to be processed entirely on the GPU through vertex and fragment shaders, reducing CPU–GPU data transfer to a minimum. MolStar’s architecture exploits this by streaming compressed coordinate data and reconstructing geometry on the GPU, achieving rendering of assemblies with over 10^6^ atoms at interactive frame rates on commodity hardware [64]. Complementary algorithmic advances, including screen-space ambient occlusion (SSAO) for depth perception [66] and LOD rendering for large assemblies [40], have further extended the visual quality and scalability of web-based viewers. The MSMS reduced-surface algorithm [67], with O(n log n) complexity, enabled real-time display of solvent-excluded surfaces for proteins of thousands of residues, a capability now standard in virtually all molecular graphics platforms.

Another interesting web-based molecular graphics tool is Protein Imager, a software oriented toward high-quality figure generation, and designed to be accessible to users without deep structural graphics expertise [68]. While it is not as widely cited as MolStar, it is mentioned in the context of web molecular viewers as a tool for streamlined visualizations. Protein Imager enables users to upload or link PDB files (or fetch them via standard PDB identifiers), choose among molecular representations (cartoons, sticks, surfaces), apply color schemes, adjust lighting/shading, and export high-resolution renderings or movie frames for publication. It tends to offer “one-click” defaults and presets geared toward producing consistent and esthetically pleasing images with minimal parameter tweaking (Fig. 6B). One of the main design philosophies of Protein Imager is to reduce user friction: no installation, no plugin, and the interface guides users toward good defaults, making it attractive for students, outreach, or quick figure preparation. Since the heavy rendering work is offloaded to the server (or handled with optimized rendering pipelines), client requirements are modest. However, the reliance on server-side rendering may introduce latency or limits in interactivity, especially with very large structures.

Additional alternatives include iCn3D [69] and MolMil [70]. iCn3D is a web-based viewer developed by NCBI that synchronizes 3D structure, 2D interaction maps, and 1D sequence/annotation tracks. The original paper [69] describes its initial capabilities, and a later expansion highlights richer analysis features and batch scripting support [71]. In iCn3D, selections in any of the views (1D, 2D, 3D) are synchronized, annotations (domains, SNPs, mutation effects) can be visualized as tracks, and custom views can be shared via permanent shortened URLs. As of its evolution, iCn3D also supports RESTful APIs, export of PNGs, batch operation through Node.js or Python integration, electrostatic potential rendering via DelPhi®, structure alignments, and expanded scripting for large datasets. The trade-off is that while iCn3D emphasizes integrated annotation and shareable state URLs, its rendering engine is less focused on extreme large-scale molecular scenes. Molmil is another web-based viewer [70] that supports on-the-fly loading of various formats and rendering of large structures, having an extendable code and a workflow that does not require data uploading to remote servers. While its user community is smaller than MolStar or iCn3D, it is a viable lightweight alternative for the integration of molecular visualizations into web sites.

## Visualization and analysis of molecular dynamics simulations

The visualization and analysis of MD trajectories are essential for converting raw simulation data into mechanistic understanding of biomolecular function, conformational dynamics, and intermolecular interactions. As modern MD simulations routinely generate terabytes of trajectory data, spanning microsecond to millisecond timescales and involving millions of atoms, the ability to interactively visualize and interrogate these datasets becomes essential. User-friendly, flexible graphical interfaces are particularly valuable because they allow researchers to efficiently filter, query, and represent specific molecular events—such as ligand binding, conformational transitions, or allosteric communication—without requiring extensive scripting. Beyond qualitative exploration, state-of-the-art visualization platforms integrate quantitative analysis modules, supporting statistical evaluations of structural fluctuations, secondary structure persistence, solvent organization, and free energy surfaces. The fusion of interactivity, computational efficiency, and extensibility thus defines the next generation of molecular graphics systems for simulation analysis, making them indispensable tools in computational biophysics and structural bioinformatics.

**Figure 7:**
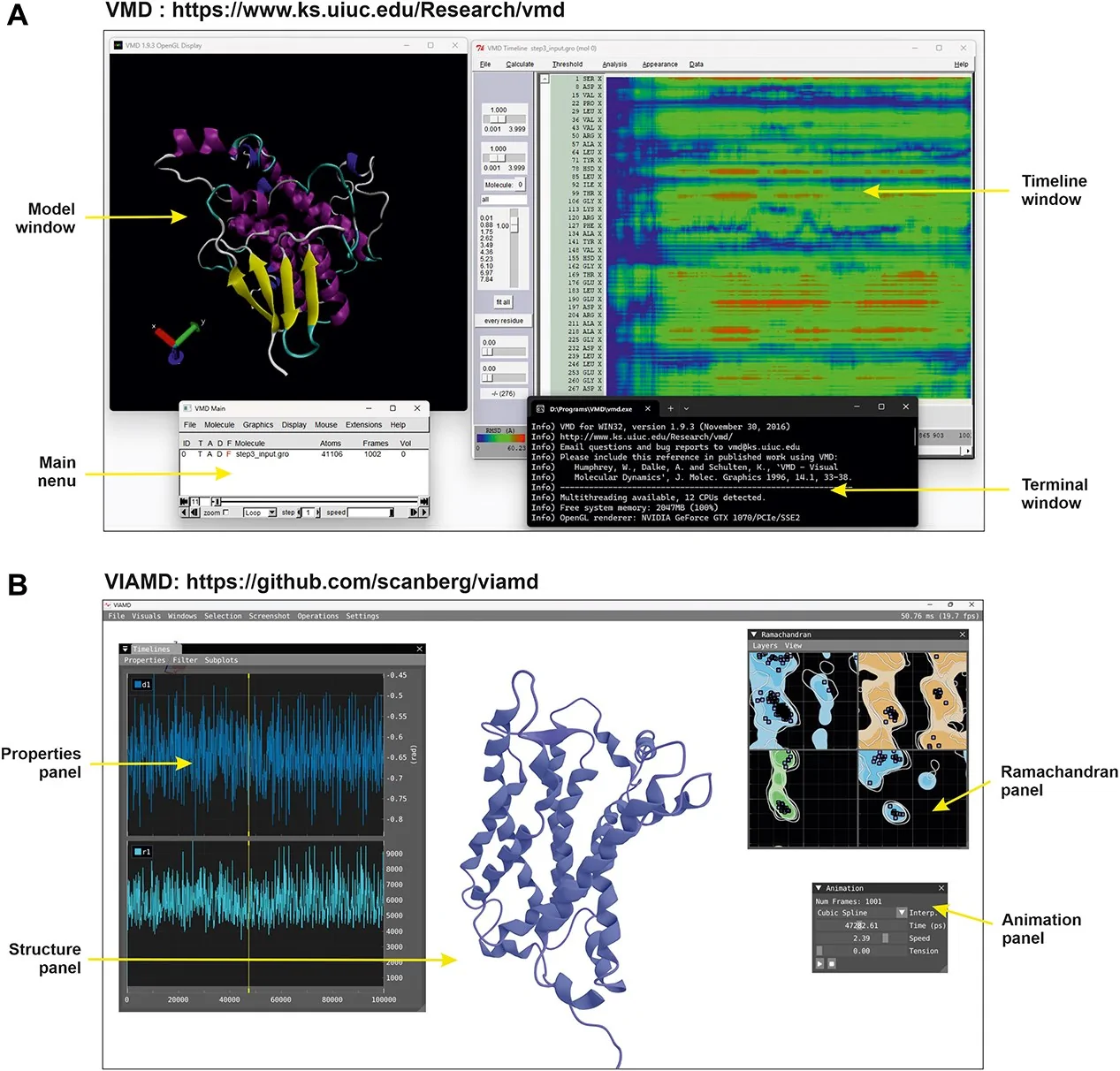
Graphical interfaces of VMD and VIAMD software used for MD trajectory analysis. (A) Screenshot of VMD v. 1.8.3 (visual molecular dynamics), a widely used open-source software for visualization and analysis of MD simulations. The interface shows the model window (left) for interactive molecular rendering, the timeline window (right) for temporal analysis of structural properties, the main menu (bottom left) for data and display management, and the terminal window (bottom right) that allow a command line interaction with the software. (B) Screenshot of VIAMD (visual interactive analysis of molecular dynamics), an interactive MD analysis environment developed at the PDC Center for High Performance Computing (KTH, Stockholm). The interface integrates several coordinated panels: the properties panel (left) displaying quantitative trajectory data; the structure panel (center) for 3D molecular visualization; the Ramachandran panel (upper right) for torsional angle distribution analysis; and the animation panel (lower right) for trajectory playback control.

Visual molecular dynamics (VMD), developed by the Theoretical and Computational Biophysics Group at the University of Illinois at Urbana–Champaign, remains one of the most comprehensive environments for trajectory analysis and molecular visualization [72]. Written primarily in C/C++ and Tcl/Tk, VMD supports an extensive array of molecular formats (PDB, DCD, PSF, GRO, XTC, TRR, AMBER NetCDF, among others) and interfaces natively with major simulation packages such as NAMD [73], GROMACS [74], CHARMM [75], and AMBER [76]. Its modular architecture integrates multiple analytical extensions capable of computing root mean square deviation (RMSD), fluctuation (RMSF), radial distribution functions, dynamic cross-correlation matrices, and time-dependent hydrogen-bond networks (Fig. 7A). In addition, VMD offers electrostatic potential calculations via the APBS plugin, volumetric density analysis, and molecular surface generation through the MSMS algorithm. A distinctive advantage of VMD lies in its powerful scripting capabilities: both Tcl and Python interfaces allow the automation of complex analyses, batch processing of large datasets, and even the development of custom graphical plugins [77]. The software also supports GPU acceleration for trajectory rendering and volumetric data processing, enabling smooth performance with very large systems. The quantitative impact of this GPU acceleration is well documented: GPU-accelerated radial distribution function analysis in VMD runs 92 times faster than an equivalent multithreaded CPU implementation, enabling analysis of atom selections containing 1 million atoms in under 27 seconds per frame [78]. GPU-accelerated electrostatic calculations achieve 10–100-fold speedups over optimized CPU implementations [79], translating directly into the ability to analyze trajectories from petascale simulations containing tens to hundreds of millions of atoms. For immersive visualization, VMD is compatible with stereoscopic displays, virtual reality systems, and remote rendering, making it a versatile solution for both research and education.

Visual interactive analysis of molecular dynamics (VIAMD), on the other hand, represents a modern, high-performance approach emphasizing interactive and visually guided exploration. Developed at the PDC Center for High Performance Computing (KTH Royal Institute of Technology, Stockholm), VIAMD is implemented in C/C++ and designed to handle long trajectories efficiently by streaming data in real time rather than loading entire files into memory [80]. It employs a lightweight internal scripting language that enables users to declare custom analytical operations—such as pairwise distance evaluations, energy decomposition, or time-dependent clustering—executed dynamically over trajectory frames (Fig. 7B). VIAMD’s interface exposes multiple synchronized visualization windows, allowing parallel analysis of structural, energetic, and spatial parameters, and supports real-time selection and manipulation of molecular subsets. The system’s efficient data management and multithreading capabilities make it particularly suited for interactive visualization of simulations generated on high-performance computing platforms.

When compared, VMD provides unmatched analytical depth, extensive plugin support, and a mature scripting ecosystem, making it ideal for advanced users conducting large-scale, automated studies or developing new visualization methodologies [81]. VIAMD, while more recent and less widespread, offers an agile, visually driven environment optimized for speed, responsiveness, and exploratory analysis, minimizing the barrier to entry for non-specialist users. Both applications, however, share the overarching goal of enabling scientists to perceive, quantify, and communicate molecular motion effectively. Together, they illustrate complementary paradigms in MD visualization—VMD as a feature-rich analytical framework with a vast user community, and VIAMD as a streamlined, next-generation platform emphasizing real-time interactivity and visual analytics for modern molecular simulation workflows.

## Virtual, extended, and immersive reality tools in molecular visualization

In recent years, immersive visualization has emerged as a powerful complement to traditional molecular graphics, allowing researchers to explore biomolecular structures in three dimensions with an unprecedented sense of spatial awareness and interaction. Among the most advanced tools developed for this purpose are UnityMol [82], VisionMol [83], Nanome [84], and VRmol [85], which represent distinct technological and conceptual approaches to virtual-reality-based molecular visualization.

UnityMol is an open-source application built on the Unity engine and programmed in C#, conceived primarily as a research platform for molecular graphics and collaborative visualization. It provides a rich suite of representations, including classical stick, cartoon, and surface models as well as the HyperBalls system for smooth atom-bond rendering [82]. The program integrates analytical tools such as molecular-dynamics trajectory visualization, electrostatic potential mapping, and Python scripting interfaces for reproducible workflows. A distinctive feature of UnityMol is its support for immersive virtual-reality sessions using SteamVR-compatible headsets such as Oculus Quest®, HTC Vive®, or Windows Mixed Reality® devices. It has been actively developed by the Baaden laboratory as a testbed for FAIR (Findable, Accessible, Interoperable, Reusable) “visualization experiences,” enabling collaborative inspection of biomolecular data within shared 3D scenes [82]. The main strengths of UnityMol lie in its openness, cross-platform compatibility (Windows, macOS, and Linux), and its extensibility for educational and research purposes [86]. Nevertheless, because it remains primarily a research-driven project, users may occasionally encounter usability inconsistencies or require familiarity with Unity scripting to take full advantage of its flexibility. The software is distributed freely under the LGPL-3.0 license, ensuring unrestricted academic use while allowing commercial adaptations under specific agreements.

VisionMol, by contrast, represents a more recent development specifically optimized for standalone and PC-based VR headsets [83]. Also constructed in Unity and programmed in C#, VisionMol provides an intuitive environment for immersive exploration of protein structures. It allows users to manipulate molecular models—rotating, scaling, and translating them through gestures or VR controllers—while offering essential analytical functionalities such as multi-model visualization, distance measurements between atoms or residues, molecular alignment, and docking evaluation. The application supports both high-end PC-VR setups and standalone devices such as the Rhino X Pro® and Meta Quest 3/Pro®, combining mobility with graphical performance. VisionMol’s principal advantage lies in its lightweight design, which makes it particularly suitable for teaching, outreach, and quick inspection of structural data without complex configuration [83]. However, as an emerging platform, its ecosystem and scripting extensibility remain limited compared with UnityMol or ChimeraX, and its analytical depth is intentionally focused on visualization rather than quantitative computation. The developers have made the source code publicly available via GitHub, suggesting a free academic model, although the licensing terms are not yet formally standardized.

**Figure 8:**
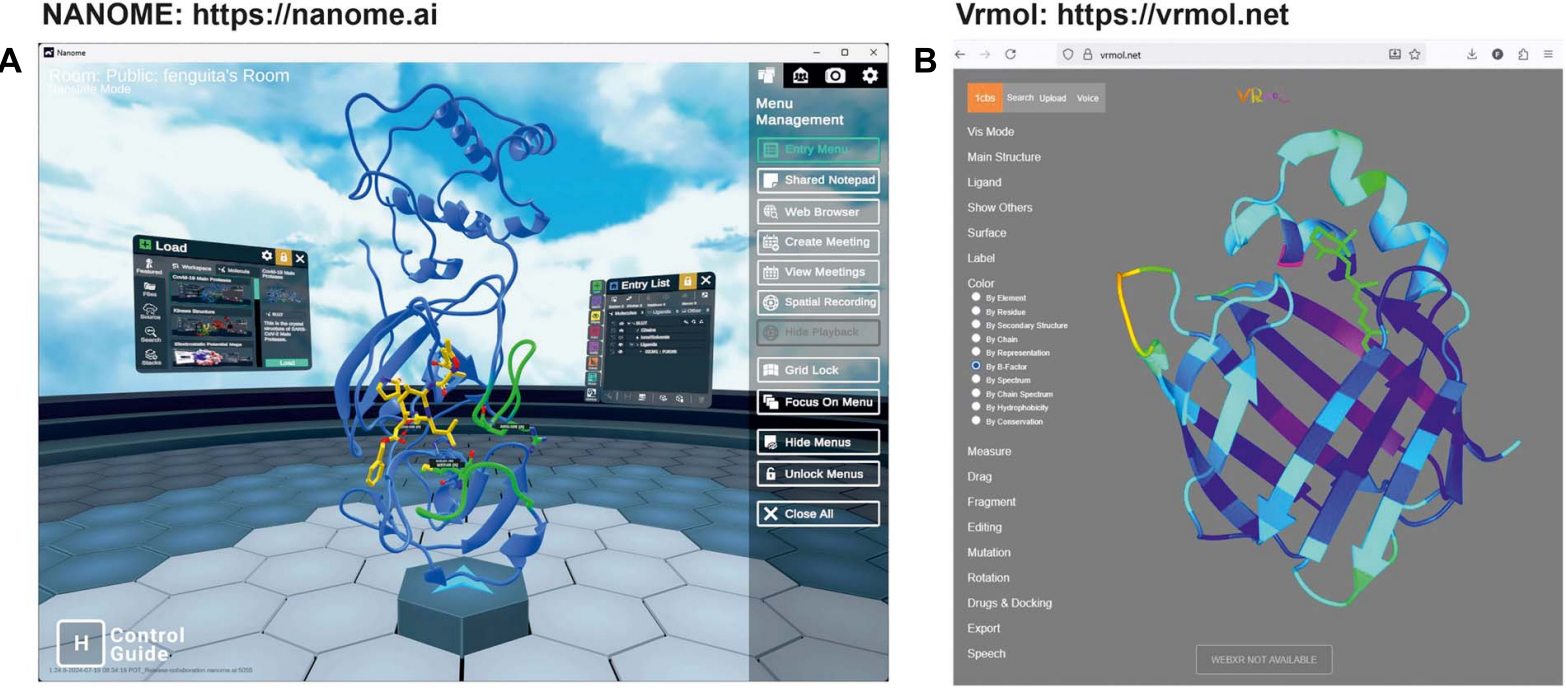
Virtual reality-based molecular visualization using VRmol and Nanome platforms. (A) Screenshot from Nanome, a commercial virtual reality application built on the Unity engine, offering an advanced, collaborative environment for molecular modeling and analysis. Nanome allows multiple users to interact with complex biomolecular systems in real time, supporting ligand docking, structural annotation, and integrated meeting tools for scientific collaboration. (B) Screenshot from VRmol, a free, web-based molecular visualization system designed for immersive exploration of biomolecular structures through standard browsers supporting WebVR/WebXR. VRmol integrates structure manipulation, coloring by various molecular attributes, and supports measurement, mutation, and docking functionalities without requiring local installation.

A different philosophy underpins Nanome, a commercial, fully integrated extended-reality environment (XR-reality), tailored for collaborative molecular modeling and drug-design workflows [84]. Nanome provides a highly polished virtual workspace in which multiple users can co-locate, examine structures, edit ligands, and analyze interactions in real time. It supports data import from resources such as the Protein Data Bank [87], PubChem [88], and DrugBank [89], and offers advanced modules for molecular editing, docking, and energy minimization. Due to its strong emphasis on remote collaboration, Nanome has become popular in both academic and industrial settings for distributed medicinal-chemistry meetings, classroom instruction, and design reviews (Fig. 8A). The platform runs on Windows®-based VR systems through SteamVR® as well as on standalone headsets such as the Meta Quest®, combining high visual fidelity with network synchronization. Its main strengths are usability, stability, and real-time collaborative capacity, which are complemented by robust enterprise support. However, the proprietary nature of the software limits custom extension, and several advanced features require paid licenses. Nanome employs a tiered commercialization strategy, with a limited free version for individual users, academic subscriptions, and enterprise packages that can exceed several thousand dollars per year, reflecting its positioning as a professional collaboration tool rather than a research prototype [84].

Finally, VRmol adopts a radically different, web-based paradigm. Developed as a WebXR/WebGL application, VRmol enables immersive molecular visualization directly within a standard browser, requiring no installation or specialized computing resources. Users can load macromolecular structures, visualize electron-density maps, or interact with integrated databases of mutations, ligands, and drugs [85]. It also supports basic molecular docking tasks executed on cloud servers. Since it runs entirely within a browser, VRmol is compatible with a wide range of devices, including desktop computers, laptops, and standalone headsets such as Oculus Quest® or HTC Vive®, provided they support a modern Web XR-enabled browser (Fig. 8B). The main advantages of VRmol are its accessibility, cross-platform nature, and integration of molecular and pharmacological databases, which make it ideal for teaching environments and for rapidly sharing interactive scenes across institutions. Nonetheless, performance and rendering fidelity are constrained by browser-based graphics and network latency, and customization options are more limited compared with native applications. VRmol is freely accessible online, with its source and associated documentation openly available, making it one of the most democratizing tools in the current VR visualization landscape [85].

Together, these four applications illustrate the breadth of approaches now available for immersive molecular visualization—from the open, scriptable framework of UnityMol, through the streamlined and portable interface of VisionMol, to the enterprise-grade collaborative environment of Nanome, and the universally accessible web-based system of VRmol. Each embodies a different balance between openness, performance, analytical capacity, and ease of use, reflecting the rapid evolution of virtual-reality technologies in structural biology and their growing integration into both research and education. The practical utility of immersive VR for molecular tasks has been quantitatively assessed: in a controlled study, participants using a VR environment completed complex 3D molecular manipulation tasks significantly faster than users working with conventional 2D interfaces, particularly for tasks whose conformational choreography is intrinsically three-dimensional [90]. In educational settings, 85% of students reported increased interest in biomolecular structures after VR-based exercises using Nanome [84].

## Conclusions and future perspectives

The historical trajectory of molecular visualization—from mechanical wire models and optical comparators to immersive, multiuser virtual environments—mirrors the technological evolution of structural biology itself. Each generation of visualization software has both reflected and propelled changes in how scientists interpret atomic data, transforming structural information into conceptual understanding (Supplementary Table S1). Early mechanical and vector-based systems established the foundations of spatial reasoning in macromolecular science; the advent of interactive graphics with Levinthal’s “Kluge” and Evans & Sutherland workstations introduced real-time molecular manipulation; and subsequent software such as FRODO, O, and RasMol democratized access to molecular representations across laboratories and classrooms.

Modern integrated platforms like PyMOL, ChimeraX, and YASARA extend this lineage by merging high-fidelity rendering with computational analysis, scripting, and automation. Parallel developments within the CCP4 ecosystem have ensured that crystallographic visualization remains tightly coupled with model building and validation, preserving reproducibility and accuracy. The emergence of web-based tools such as Mol*, Protein Imager, and iCn3D represents another paradigm shift, promoting universal accessibility, cloud integration, and collaborative science. More recently, virtual and extended reality frameworks, including UnityMol, VisionMol, Nanome, and VRmol, have brought molecular visualization into immersive, interactive, and even social dimensions, bridging the gap between research, education, and public engagement.

Looking ahead, several converging trends are poised to define the next era of molecular visualization. The exponential growth of structural data—fueled by cryo-electron microscopy, integrative modeling, and AI-based structure prediction (e.g. AlphaFold and ESM)—demands scalable visualization architectures capable of rendering and analyzing entire proteomes or cellular environments in real time. Integration with artificial intelligence will enable adaptive visualization, in which salient features such as binding pockets, conformational transitions, or conserved surfaces are automatically highlighted and interpreted. Cloud computing and web technologies will further enhance collaboration by enabling synchronous multiuser sessions and persistent, shareable 3D workspaces. Likewise, extended-reality systems are expected to converge with haptic and gestural interfaces, offering natural modes of molecular manipulation that transcend traditional input devices.

Despite these advances, key challenges remain. The balance between visual realism and scientific accuracy must be preserved to avoid esthetic distortion of molecular data. Standardization of file formats, APIs, and metadata is essential to ensure interoperability among tools and databases. Moreover, as visualization environments increasingly integrate simulation, docking, and AI prediction, transparency of algorithms and reproducibility of visual analyses must be prioritized within FAIR data principles.

Ultimately, molecular visualization has evolved from a specialized aid for crystallographers into a universal language of structural biology—an indispensable interface between computation and intuition. Its continuing development will not only accelerate discovery but also redefine how molecular science is communicated, taught, and experienced. By tracing its past and envisioning its future, we recognize molecular graphics as both a technological heritage and a living frontier that continues to expand the cognitive and creative reach of the molecular sciences.

**Key Points**

- Provide a historical narrative of molecular visualization, tracing its evolution from physical models and early optical devices to modern GPU-accelerated and immersive digital tools.
- Highlights major technological milestones and foundational platforms—including Levinthal’s “Kluge,” Evans & Sutherland hardware, FRODO, RasMol, PyMOL, ChimeraX, VMD, and CCP4-based environments—that shaped structural biology workflows.
- Describe how visualization software evolved from descriptive tools to integrative analytical environments, incorporating simulation, model building, docking, and scripting.
- Examine the rise of web-based and immersive VR/AR platforms, such as MolStar, Nanome, UnityMol, and VRmol, which broaden access and enable collaborative, multiuser molecular exploration.
- Discuss future directions including AI-assisted visualization, cloud-based collaboration, and scalable rendering for structural data.

## Supplementary Material

Supplementary_table_1_v2_bbag373

## Data Availability

This article is a review and does not involve the generation of new datasets. All information presented is derived from previously published literature and publicly available software resources. The software tools discussed in this review can be accessed through their respective official repositories or project websites, as cited throughout the manuscript.
