# Supplementary material for: From wireframes to immersive reality: a historical perspective on molecular visualization software
Source: Brief Bioinform. 2026 Sep 7;27(5):bbag373. doi: 10.1093/bib/bbag373 (PMC13549435; doi:10.1093/bib/bbag373)
Supplement: Supplementary file 1 [file supplementary_table_1_v2_bbag373.docx]

| **Software** | **OS** | **Distribution** | **Web** | **Comments** | **Ref.** |
| --- | --- | --- | --- | --- | --- |
| **Avogadro** | Linux, MacOS and Windows | Freeware | https://avogadro.cc | Avogadro is an open-source, cross-platform molecular editor and visualization program designed for computational chemistry, molecular modeling, and materials science applications. It provides an intuitive graphical interface that allows users to build, manipulate, and analyze molecular structures with support for a wide variety of file formats, including PDB, MOL, CML, and Gaussian input/output files.  **Advantages:**   - Intuitive and user-friendly graphical interface for molecular building and visualization. - Open-source and cross-platform (Windows, macOS, Linux). - Supports a wide range of chemical file formats (e.g., PDB, MOL, CML, Gaussian).   **Limitations:**   - Performance constraints with very large or highly complex molecular systems. - Reliance on external software for high-level computational workflows. - Fewer specialized features compared to high-end commercial packages. | [1] |
| **BIOVIA Discovery Studio** | Linux and Windows | Freeware for BIOVIA Discovery Studio Visualizer and commercial license for other modules | https://www.3ds.com/products/biovia | BIOVIA Discovery Studio is a comprehensive molecular modeling and simulation platform widely used in drug discovery and structural biology. It integrates tools for sequence analysis, homology modeling, docking, molecular dynamics, and free energy calculations within a single environment. Key features include flexible protein and ligand modeling, CHARMm- and NAMD-based simulations with enhanced sampling methods and docking workflows for protein–ligand and protein–protein interactions. The suite also provides predictive tools for biologics design, such as antibody modeling and aggregation assessment, along with high-quality visualization and a free Discovery Studio Visualizer for data sharing.  **Advantages:**   - Comprehensive, integrated platform covering sequence analysis, modeling, docking, molecular dynamics, and free energy calculations with high-quality visualization and specialized tools for biologics design   **Limitations:**   - Commercial licensing costs, high computational resource requirements, and reduced flexibility/customization compared to open-source or script-based workflows | [2] |
| **BioViz Studio** | Web-based platform independent | Commercial license | https://www.bioviz-studio.com | BioViz Studio is a web-based molecular animation platform designed to let researchers convert structural models into polished, scientifically accurate videos without needing local 3D-rendering software. Users can import molecular data (e.g. PDB, mmCIF, MOL2, SDF) either from local files or direct retrieval from the RCSB PDB, and then assemble multi-molecule scenes with independent transformations, representation styles, and animated parameters.  **Advantages:**   - User-friendly, web-based platform enabling rapid creation of high-quality molecular animations with no need for local rendering software and easy integration of structural data from multiple sources   **Limitations:**   - Limited control over advanced rendering and simulation features, dependence on internet connectivity, and potential constraints in handling very large or highly complex molecular scenes | [3] |
| **CCP4mg** | Linux, MacOS and Windows | Freeware | https://www.ccp4.ac.uk/MG | CCP4mg is a molecular graphics program developed within the Collaborative Computational Project No. 4 (CCP4) suite, specifically designed to support macromolecular crystallography and structural biology research. The software provides an integrated environment for the visualization of atomic models, electron density maps, and related structural data, enabling detailed analysis of crystallographic results.  **Advantages:**   - Specialized for macromolecular crystallography with strong support for electron density map visualization and model validation. - Seamless integration with the CCP4 suite, facilitating crystallographic workflows. - High-quality graphical representations tailored for structural analysis and publication. - Supports multiple structural data formats commonly used in crystallography.   **Limitations:**   - Less intuitive interface compared to modern molecular viewers. - Limited functionality outside crystallography-focused applications. - Not designed for advanced molecular dynamics or quantum simulations. - Smaller plugin ecosystem and customization options compared to more general-purpose tools. | [4] |
| **Coot** | Linux, MacOS and Windows | Freeware | https://www2.mrc_lmb.cam.ac.uk/personal/pemsley/coot | Coot (Crystallographic Object-Oriented Toolkit) is an open-source software package specifically developed for the visualization, building, and validation of macromolecular models in structural biology. It is widely used in crystallography and cryo-electron microscopy workflows, where it provides an interactive environment for fitting atomic models into experimental electron density or cryo-EM maps. Coot supports real-space refinement, torsion and geometry restraints, and automated tools for side-chain fitting, rotamer selection, and ligand placement, enabling accurate model improvement during structure determination.  **Advantages:**   - Open-source and widely adopted for macromolecular model building and validation in crystallography and cryo-EM. - Powerful interactive tools for real-space refinement, map fitting, and geometry correction. - Efficient handling of electron density and cryo-EM maps with robust validation features. - Automated functionalities for side-chain fitting, rotamer selection, and ligand placement.   **Limitations:**   - Primarily focused on model building rather than full simulation or predictive modeling. - Interface can be less intuitive for new users compared to modern visualization tools. - Dependence on external software for complete structural determination workflows. | [5] |
| **Ezmol** | Web-based platform independent | Freeware | https://www.sbg.bio.ic.ac.uk/~ezmol | EZMol is a browser-based molecular visualization tool designed to provide a simple and intuitive interface for exploring protein and nucleic acid structures without requiring installation or advanced expertise. The platform focuses on ease of use, offering a streamlined workflow where users can upload PDB files and rapidly generate high-quality molecular images.  **Advantages:**   - Simple, intuitive web-based interface requiring no installation or advanced expertise. - Rapid generation of high-quality molecular images suitable for teaching and publications. - Accessible from any platform with minimal setup.   **Limitations:**   - Limited functionality compared to full-featured molecular modeling or analysis tools. - Restricted customization and lack of advanced scripting or automation options. - Not suitable for large-scale systems or complex structural analyses. | [6] |
| **ICM Suite** | Linux, MacOS and Windows | Freeware for ICM-browser, commercial for the other components of the suite | https://www.molsoft.com | The ICM (Internal Coordinate Mechanics) software suite is a comprehensive platform for molecular visualization, modeling, and computational drug discovery, widely used in structural biology and cheminformatics. It combines high-performance molecular graphics with advanced modeling algorithms, enabling interactive visualization of proteins, nucleic acids, ligands, and complexes in a flexible 3D environment. The suite supports a broad range of functionalities, including homology modeling, flexible molecular docking, binding pocket identification, structure-based virtual screening, and quantitative structure–activity relationship (QSAR) analysis.  **Advantages:**   - Comprehensive platform integrating visualization, docking, virtual screening, and QSAR within a single environment. - Efficient internal coordinate representation enabling fast conformational sampling and flexible docking. - Strong performance in structure-based drug design and binding site analysis.   **Limitations:**   - Commercial licensing costs and restricted accessibility. - Steep learning curve due to specialized workflows and scripting environment. - Limited transparency/customization compared to open-source alternatives. | [7] |
| **iCn3D** | Web-based platform independent | Freeware | https://www.ncbi.nlm.nih.gov/Structure/icn3d | iCn3D is a web-based molecular visualization tool developed by the National Center for Biotechnology Information (NCBI) that integrates with the AlphaFold database, Molecular Modeling Database (MMDB), Protein Data Bank (PDB), and other NCBI resources. It allows interactive three-dimensional visualization of biomolecular structures directly in a browser supporting a wide range of representations such as ribbons, sticks, spheres, molecular surfaces, and electrostatic potentials.  **Advantages:**   - Web-based and freely accessible with no installation required, enabling easy sharing and collaboration. - Seamless integration with NCBI resources (MMDB, PDB, AlphaFold) for rapid data retrieval and analysis - Interactive visualization with multiple. representation styles and annotation features.   **Limitations:**   - Limited advanced modeling, simulation, and editing capabilities compared to standalone software. - Performance may be constrained by very large or complex structures. - Dependence on internet connectivity and browser compatibility. | [8] |
| **iMolView** | Android and iOS for mobile devices | Freeware | https://www.molsoft.com/iMolview.html | iMolview is a molecular visualization and modeling application optimized for mobile devices, designed to make structural biology and cheminformatics tools accessible on smartphones and tablets. The software enables interactive 3D visualization of proteins, nucleic acids, ligands, and small molecules, with intuitive touch-based controls for rotation, zooming, and structural manipulation. Users can directly access and download structural data from the Protein Data Bank (PDB) or chemical databases such as PubChem, allowing seamless integration of biomolecular and chemical information.  **Advantages:**   - Mobile-optimized interface with intuitive touch controls for interactive 3D visualization. - Direct access to structural and chemical databases (PDB, PubChem) for quick data retrieval. - Portable and convenient for teaching, presentations, and on-the-go exploration.   **Limitations:**   - Limited functionality compared to desktop-based molecular modeling tools. - Performance and rendering constraints for large or complex structures. - Restricted advanced analysis, simulation, and customization capabilities. | [9] |
| **jMol** | Java-based platform independent | Freeware | https://jmol.sourceforge.net | Jmol is an open-source molecular visualization and modeling program that provides a versatile platform for interactive exploration of biomolecular and chemical structures. Implemented in Java, it runs on multiple operating systems and is also available as JSmol, a browser-based variant that requires no plugins, ensuring wide accessibility. Jmol supports a broad range of file formats, including PDB, CIF, MOL, and SDF, enabling visualization of proteins, nucleic acids, small molecules, and crystallographic data.  **Advantages:**   - Open-source and highly portable, with both desktop (Java-based) and browser-based (JSmol) versions. - Supports a wide range of molecular and crystallographic file formats. - Flexible scripting capabilities for customized visualization and teaching applications.   **Limitations:**   - Outdated interface and graphics compared to modern visualization tools. - Limited advanced modeling and simulation features. - Performance and usability may be constrained for very large or complex systems. | [10] |
| **Mol* (MolStar)** | Web-based platform independent | Freeware | http://molstar.org | Mol* is an open-source, browser-based platform designed for interactive visualization and analysis of macromolecular structures and related experimental datasets. Built on WebGL technology, it delivers high-performance rendering directly online, supporting efficient streaming of structural coordinates and volumetric information.  **Advantages:**   - High-performance WebGL-based rendering enabling smooth visualization of very large macromolecular structures directly in the browser. - Efficient streaming and handling of structural and volumetric data (e.g., electron density, cryo-EM maps). - Open-source and easily shareable, supporting collaborative and web-integrated workflows.   **Limitations:**   - Limited capabilities for advanced modeling, editing, or simulation compared to desktop applications. - Dependence on browser performance and internet connectivity for optimal use. - Less flexible for highly customized or script-intensive workflows. | [11] |
| **Molecular Operating Environment (MOE)** | Linux, MacOS and Windows | Commercial license | https://www.chemcomp.com/en/Products.htm | The Molecular Operating Environment (MOE) is a comprehensive, commercial software platform developed by the Chemical Computing Group that integrates molecular visualization, modeling, and computational chemistry tools into a single environment. Designed to support both academic research and pharmaceutical discovery, MOE provides a wide range of capabilities including protein and ligand structure preparation, homology modeling, molecular docking, and quantitative structure–activity relationship (QSAR) modeling.  **Advantages:**   - Comprehensive, integrated platform covering structure preparation, modeling, docking, and QSAR within a single environment. - User-friendly interface with robust workflows for drug discovery and structural biology applications. - Well-validated algorithms and reliable performance for both academic and industrial research.   **Limitations:**   - Commercial licensing costs may limit accessibility. - Less flexible and customizable compared to open-source or scripting-based tools. - High computational demands for advanced simulations and large-scale analyses. | N/A |
| **Molecular Rift** | Linux | Freware | https://github.com/JBostrom/MolecularRiftv2 | Molecular Rift is a virtual reality (VR) application developed to explore and manipulate molecular structures in an immersive environment using head-mounted displays such as the Oculus Rift. It provides an intuitive interface where users can naturally interact with biomolecules by grabbing, rotating, and scaling them in three-dimensional space, thereby enhancing spatial understanding of complex structural features.  **Advantages:**   - Immersive VR environment enabling intuitive, hands-on interaction with molecular structures. - Enhances spatial understanding of complex biomolecular architectures. - Engaging tool for education, outreach, and collaborative exploration.   **Limitations:**   - Requires specialized VR hardware (e.g., Oculus Rift), limiting accessibility. - Limited functionality for advanced analysis, modeling, or simulation workflows. - Potential performance constraints and usability challenges for extended sessions. | [12] |
| **MolecularWeb** | Web-based platform independent | Freware | https://molecularweb.epfl.ch | MoleculARweb is an open-access educational platform that brings molecular visualization into classrooms through interactive augmented reality (AR) and virtual reality (VR) tools. Designed to run directly in a web browser without the need for installation, it uses device cameras to recognize printable AR markers, which then display three-dimensional, interactive models of biomolecules such as proteins, nucleic acids, and small ligands.  **Advantages:**   - Free, web-based platform requiring no installation, easily accessible for classroom use. - Interactive AR/VR visualization enhances engagement and understanding of molecular structures. - Simple setup using printable markers and standard devices (e.g., smartphones, tablets, webcams).   **Limitations:**   - Limited functionality for advanced analysis, modeling, or research-grade workflows. - Dependence on device camera quality and environmental conditions for accurate AR tracking. - Less suitable for handling large or highly complex molecular systems. | [13] |
| **MolMil** | Web-based platform independent | Freeware | http://gjbekker.github.io/molmil | Molmil is an advanced browser-based molecular graphics system created by the Protein Data Bank Japan (PDBj) to provide efficient, interactive visualization of structural data without the need for additional plugins. Leveraging WebGL and GPU acceleration, it can display very large molecular complexes, such as ribosomes and viral assemblies, with smooth performance. The viewer accommodates common coordinate and density formats, including PDB, mmCIF, and volumetric maps, and offers flexible display modes ranging from atomic sticks and space-filling models to ribbons, surfaces, and electrostatic overlays.  **Advantages:**   - High-performance WebGL and GPU-accelerated rendering enabling smooth visualization of very large macromolecular complexes. - Browser-based with no plugins required, allowing easy access and sharing. - Supports multiple structural and volumetric formats with versatile representation options.   **Limitations:**   - Limited advanced modeling, editing, and simulation capabilities compared to desktop tools. - Dependence on browser and hardware performance for optimal rendering. - Less flexible for scripting and highly customized workflows. | [14] |
| **Nanome** | Windows | Free limited personal license and commercial license for expanded capabilities | https://nanome.ai | Nanome (Nanome.ai) is a virtual reality (VR) platform designed to bring molecular modeling and structural analysis into immersive, spatial computing environments. It enables researchers and students to load biomolecular structures (e.g. proteins, ligands, nucleic acids), electron density maps, and trajectories, then explore and manipulate them in three dimensions using natural gestures in VR.  **Advantages:**   - Immersive VR environment enabling intuitive, spatial interaction with molecular structures and datasets. - Supports collaborative, real-time exploration and manipulation of biomolecules and trajectories. - Effective for enhancing structural insight in research, education, and drug design workflows.   **Limitations:**   - Requires specialized VR hardware, limiting accessibility and increasing cost. - Limited advanced simulation capabilities compared to traditional computational chemistry platforms. - Potential performance and usability constraints for very large systems or prolonged sessions. | [15] |
| **Protein Imager** | Web-based platform independent | Open Access | https://3dproteinimaging.com | Protein Imager is a web-based molecular visualization platform developed to provide high-quality, interactive 3D representations of biomolecular structures directly in a browser environment. Designed with accessibility in mind, it does not require local installation and allows users to upload their own coordinate files or fetch entries directly from the Protein Data Bank (PDB). The software offers a variety of visualization modes, along with customizable coloring schemes to emphasize secondary structure elements, chains, ligands, or functional motifs. Protein Imager also supports advanced rendering features, such as transparency, lighting effects, and depth cues, enabling the generation of publication-ready figures.  **Advantages:**   - Web-based and accessible with no installation required, enabling easy use and sharing. - High-quality rendering with customizable visualization styles suitable for publication-ready figures. - Flexible input options, including direct PDB retrieval and user-uploaded structures.   **Limitations:**   - Limited capabilities for advanced modeling, simulation, or structural editing. - Performance may be constrained for very large or complex systems. - Dependence on browser performance and internet connectivity. | [16] |
| **Pymol** | Linux, MacOS and Windows | User-sponsored software under an open-source foundation | https://www.pymol.org | PyMOL is a widely used molecular visualization and modeling software that has become a standard tool in structural biology, computational chemistry, and drug discovery. It supports a broad spectrum of molecular representations, including ribbons, sticks, spheres, surfaces, and volumetric maps, allowing detailed exploration of proteins, nucleic acids, ligands, and complexes. One of its key strengths lies in producing high-resolution, publication-quality images and animations, with advanced control over lighting, shading, coloring, and transparency.  **Advantages:**   - High-quality rendering producing publication-ready images and animations with fine control over visual parameters. - Widely adopted and well-documented, with strong scripting support for automation and reproducibility. - Supports diverse molecular representations and extensive structural analysis features.   **Limitations:**   - Steeper learning curve, particularly for advanced scripting and customization. - Limited native capabilities for molecular dynamics or quantum simulations. - Some advanced features require the commercial version (Incentive PyMOL). | [17] |
| **QuteMol** | MacOS and Windows | Freeware | https://qutemol.sourceforge.net | QuteMol is an open-source molecular visualization software designed to produce high-quality, photorealistic renderings of biomolecular structures. It employs advanced real-time shading and lighting techniques, such as ambient occlusion and depth cueing, to enhance the perception of spatial relationships and structural details in complex molecules. QuteMol supports standard structural file formats, including PDB, allowing visualization of proteins, nucleic acids, ligands, and molecular assemblies.  **Advantages:**   - Produces highly photorealistic molecular renderings using advanced shading (e.g., ambient occlusion, depth cueing). - Open-source and relatively easy to use for generating visually appealing structural images. - Effective for highlighting spatial relationships and structural features in biomolecules.   **Limitations:**   - Limited functionality for analysis, editing, or advanced molecular modeling. - Largely outdated with minimal ongoing development and support. - Restricted customization and integration compared to modern visualization tools. | [18] |
| **Rasmol** | Linux, MacOS and Windows | Freeware | https://www.umass.edu/microbio/rasmol | RasMol is one of the pioneering molecular graphics programs, widely recognized for its role in making three-dimensional biomolecular visualization broadly accessible to the scientific community. As a lightweight and efficient application, RasMol supports a variety of structural file formats, including PDB, CIF, and MOL, allowing users to explore proteins, nucleic acids, small molecules, and crystallographic data.  **Advantages:**   - Lightweight and fast, enabling efficient visualization even on low-resource systems. - Historically important and widely accessible, supporting multiple standard structural formats. - Simple command-based interface allowing basic scripting and customization.   **Limitations:**   - Outdated graphics and user interface compared to modern visualization tools. - Limited functionality for advanced analysis, modeling, or high-quality rendering. - Minimal ongoing development and lack of integration with modern computational workflows. | [19] |
| **SAMSON connect** | Linux, MacOS and Windows | Freeware for starter plan and commercial license for other extended plans | https://www.samson-connect.net | SAMSON (Software for Adaptive Modeling and Simulation of Nanosystems) is a modular platform designed for interactive visualization, modeling, and simulation of molecular and nanoscale systems. Its architecture is based on a flexible framework of plug-ins, which extend the core environment with specialized tools for tasks such as molecular docking, quantum chemistry, coarse-grained modeling, or machine-learning–based property prediction. The software provides real-time, high-quality 3D rendering of biomolecules, nanostructures, and materials, with customizable representations ranging from atomic detail to supramolecular assemblies.  **Advantages:**   - Modular, plugin-based architecture allowing flexible extension with diverse modeling and simulation tools. - Interactive, real-time 3D visualization of molecular and nanoscale systems with high-quality rendering. - Supports multidisciplinary workflows, including docking, quantum chemistry, and machine learning applications.   **Limitations:**   - Many advanced features require paid plugins, limiting full functionality in the free version. - Steeper learning curve due to modular ecosystem and configuration complexity. - Performance and usability may vary depending on installed modules and system resources. | [20] |
| **Schrödinger Maestro** | Linux, MacOS and Windows | Free limited educational license and commercial license for full suite | https://www.schrodinger.com/platform/products/maestro | Schrödinger’s Maestro is the central interface of the Schrödinger Suite, designed to integrate advanced computational methods for molecular modeling, simulation, and drug discovery into a single platform. It provides a highly interactive 3D environment for building and visualizing molecular structures, with customizable representations that highlight atomic interactions, binding sites, and physicochemical properties.  **Advantages:**   - Highly integrated platform combining visualization, modeling, docking, and simulation tools within a unified interface. - Advanced, well-validated algorithms for drug discovery workflows (e.g., Glide, Desmond, Prime). - High-quality visualization and user-friendly environment tailored for both academia and industry.   **Limitations:**   - Expensive commercial licensing limiting accessibility. - High computational resource requirements for advanced simulations. - Less flexible and customizable compared to open-source or script-based solutions. | N/A |
| **Swiss PDB Viewer** | Linux, MacOS and Windows | Freeware | https://spdbv.unil.ch | Swiss-PdbViewer is a molecular visualization and modeling software developed at the Swiss Institute of Bioinformatics that provides an integrated platform for structural analysis and comparative modeling of biomolecules. It allows the visualization of proteins, nucleic acids, and complexes in multiple representations. One of its distinctive strengths is the tight integration with the SWISS-MODEL server, enabling users to perform homology modeling directly from sequence alignments to structural templates within the same environment.  **Advantages:**   - Integrated environment for structural visualization and homology modeling with direct connection to SWISS-MODEL. - User-friendly interface suitable for comparative modeling and educational purposes. - Supports multiple structural representations and basic analysis tools.   **Limitations:**   - Limited advanced features for simulation, docking, or large-scale structural analysis. - Outdated interface and reduced development activity compared to modern tools. - Less flexibility and customization compared to more recent visualization platforms. | [21] |
| **UCSF ChimeraX** | Linux, MacOS and Windows | Freeware | https://www.rbvi.ucsf.edu/chimerax | UCSF ChimeraX is a next-generation molecular visualization and analysis system developed to address the demands of modern structural biology and cryo-EM research. It excels at handling very large models and volumetric datasets with responsive rendering, including ambient occlusion and advanced lighting for enhanced depth cues. ChimeraX supports interactive fitting and segmentation of density maps, morphing between conformational states, and superposition of multiple structures for comparative analyses.  **Advantages:**   - High-performance visualization capable of handling very large structures and volumetric datasets (e.g., cryo-EM maps). - Advanced rendering (ambient occlusion, lighting) and analysis tools for fitting, segmentation, and structural comparison. - Modern interface with powerful scripting and extensibility for complex workflows.   **Limitations:**   - Steep learning curve for advanced features and command-line usage. - Limited native support for full molecular dynamics or quantum simulations. - High hardware requirements for optimal performance with large datasets. | [22] |
| **UnityMol** | Based on Unity3D engine. Runs on Linux, MacOS and Windows | Freeware | https://unity.mol3d.tech | UnityMol is a visualization environment built on the Unity 3D engine that leverages virtual reality (VR) to provide a distinctive approach to molecular analysis. Instead of relying solely on conventional screen-based inspection, UnityMol immerses users in a 3D workspace where molecular structures can be directly manipulated through intuitive actions such as reaching, scaling, or walking around the model. UnityMol also supports collaborative VR sessions, enabling multiple users to explore and annotate the same molecular system in real time, which is particularly valuable for teaching or team-based analysis.  **Advantages:**   - Immersive VR environment enabling intuitive, hands-on interaction with molecular structures. - Supports real-time collaborative sessions for shared exploration and annotation. - Enhances spatial understanding and engagement, particularly in teaching and team-based analysis.   **Limitations:**   - Requires VR hardware, limiting accessibility and increasing setup complexity. - Limited advanced modeling and simulation capabilities compared to traditional tools. - Performance and usability may vary depending on hardware and system size. | [23] |
| **ViaMD** | Linux, MacOS and Windows | Freeware | https://github.com/scanberg/viamd | ViaMD is an interactive software environment for the analysis and visualization of molecular dynamics (MD) simulations, implemented in C and C++. Developed at the PDC Center for High Performance Computing, KTH Royal Institute of Technology (Stockholm, Sweden), the program integrates computational efficiency with an intuitive graphical interface. It features a lightweight scripting language that enables users to specify analytical operations applied across trajectory frames. The resulting data can be displayed in multiple visualization panels, allowing dynamic inspection and interpretation of molecular motions and simulation-derived properties.  **Advantages:**   - Specialized for molecular dynamics analysis with efficient handling of trajectory data. - Lightweight scripting language enabling flexible and automated analysis workflows. - Interactive visualization panels for dynamic exploration of simulation results.   **Limitations:**   - Limited functionality outside MD analysis (e.g., docking, quantum calculations). - Smaller user community and ecosystem compared to widely adopted tools. - Interface and features may be less polished than more mature visualization platforms. | [24] |
| **VisionMol** | Based on Unity3D engine, developed on Windows | Freeware | https://github.com/WangLabforComputationalBiology/VisionMol | VisionMol enables immersive exploration and analysis of three-dimensional molecular structures across VR platforms (e.g., Rhino X Pro, Meta Quest Pro/3) and desktop systems. Developed in Unity with C#, it integrates custom scripts for molecular manipulation, allowing rotation, scaling, and translation via gestures or controllers. The platform supports multi-model visualization, distance measurement, and molecular alignment or docking, providing an interactive and versatile environment for structural analysis.  **Advantages:**   - Immersive VR and desktop support enabling intuitive manipulation of molecular structures. - Interactive tools for measurement, alignment, and docking within a 3D environment. - Supports multi-model visualization, enhancing comparative structural analysis.   **Limitations:**   - Requires VR hardware for full functionality, limiting accessibility. - Limited advanced simulation capabilities compared to traditional computational chemistry platforms. - Performance and usability may depend on hardware and complexity of the molecular systems. | [25] |
| **VMD** | Linux, MacOS and Windows | Freeware | https://www.ks.uiuc.edu/Research/vmd | Visual Molecular Dynamics (VMD) is a high-performance software platform designed for the visualization and analysis of large biomolecular systems, widely used in structural biology, computational chemistry, and molecular simulations. It supports an extensive range of structural and trajectory file formats, enabling the interactive exploration of proteins, nucleic acids, membranes, and molecular complexes derived from crystallography, cryo-EM, or molecular dynamics simulations. VMD offers a broad spectrum of graphical representations, including ribbons, sticks, surfaces, volumetric maps, and electrostatic fields, with advanced rendering features for generating publication-quality images and animations.  **Advantages:**   - High-performance platform capable of handling very large biomolecular systems and MD trajectories. - Extensive support for file formats and advanced visualization/analysis tools (including scripting via Tcl/Python). - Strong integration with simulation workflows and ability to generate high-quality images and animations.   **Limitations:**   - Steep learning curve, particularly for scripting and advanced analyses. - Interface can feel less modern and less intuitive for new users. - Limited native capabilities for setup of simulations or quantum-level calculations. | [26] |
| **VRMol** | Web-based platform independent | Freware | https://vrmol.net | VRmol is a cloud-based molecular visualization and analysis platform that integrates structural biology with immersive virtual reality (VR) technologies. Accessible through standard web browsers and compatible with major VR headsets, it enables interactive exploration of proteins, nucleic acids, ligands, cryo-EM maps, and molecular assemblies in a fully three-dimensional environment. VRmol supports a wide range of structural formats, including PDB and mmCIF, and incorporates functional annotations from external databases, allowing users to connect molecular structures with sequence, mutation, and functional data.  **Advantages:**   - Cloud-based and browser-accessible with optional VR support, enabling flexible and immersive molecular exploration. - Integration with external databases linking structural data to sequence, mutation, and functional annotations. - Supports multiple structural formats and interactive visualization of complex biomolecular systems.   **Limitations:**   - Dependence on internet connectivity and browser performance. - Limited advanced modeling, simulation, or editing capabilities compared to desktop tools. - VR functionality requires compatible hardware, which may limit accessibility. | [27] |
| **VTX** | Linux and Windows | Freeware | https://vtx.drugdesign.fr | VTX is a lightweight, browser-based molecular visualization platform built on WebGL, enabling fast, installation-free inspection of biomolecular structures. It supports standard formats (PDB, mmCIF) and can display molecular dynamics trajectories, offering smooth navigation of large systems. Core features include cartoon, stick, and surface representations, residue and ligand selection, color-by-property tools, and distance measurements.  **Advantages:**   - Lightweight, browser-based platform requiring no installation with fast WebGL rendering. - Supports both static structures and MD trajectories with smooth navigation of large systems. - Provides essential visualization and analysis tools (representations, selection, measurements).   **Limitations:**   - Limited advanced modeling, simulation, or editing capabilities. - Fewer customization and scripting options compared to full-featured desktop tools. - Performance dependent on browser and hardware for very large or complex datasets. | [28] |
| **YASARA** | Linux, MacOS and Windows | Freeware for YASARA view and commercial license for other modules | https://www.yasara.org/ | YASARA (Yet Another Scientific Artificial Reality Application) is a multifunctional platform that integrates molecular visualization with modeling, simulation, and drug discovery tools in a single environment. It distinguishes itself through real-time, interactive graphics that deliver photorealistic rendering of proteins, nucleic acids, membranes, and small molecules, while allowing smooth manipulation even for large systems. In addition to conventional display options, YASARA provides advanced features such as interactive mutagenesis, protonation state assignment, and automated preparation of molecular systems for simulations. A major strength of the software is its built-in molecular dynamics engine, optimized for speed on standard hardware and capable of handling long simulations with adaptive force fields.  **Advantages:**   - Integrated platform combining visualization, modeling, and built-in molecular dynamics simulations. - High-performance, photorealistic rendering with real-time interaction, even for large systems. - User-friendly tools for system preparation, mutagenesis, and simulation setup.   **Limitations:**   - Commercial licensing for full-featured versions may limit accessibility. - Less widely adopted in some research communities compared to tools like VMD or GROMACS-based workflows. - Limited flexibility/customization compared to fully open-source or script-driven environments. | [29] |

**Supplementary Table 1: alphabetic list of selected molecular graphics software.** Every piece of software includes its reference name, the operative systems (OS) where the software can be executed, the license type, web site address, operational description and references.

**References**

1.	Hanwell MD, Curtis DE, Lonie DC et al. Avogadro: an advanced semantic chemical editor, visualization, and analysis platform, J Cheminform 2012;4:17.

2.	Kemmish H, Fasnacht M, Yan L. Fully automated antibody structure prediction using BIOVIA tools: Validation study, PLoS One 2017;12:e0177923.

3.	Li H, Wei X. A Concise Review of Biomolecule Visualization, Curr Issues Mol Biol 2024;46:1318-1334.

4.	McNicholas S, Potterton E, Wilson KS et al. Presenting your structures: the CCP4mg molecular-graphics software, Acta Crystallogr D Biol Crystallogr 2011;67:386-394.

5.	Emsley P, Lohkamp B, Scott WG et al. Features and development of Coot, Acta Crystallogr D Biol Crystallogr 2010;66:486-501.

6.	Reynolds CR, Islam SA, Sternberg MJE. EzMol: A Web Server Wizard for the Rapid Visualization and Image Production of Protein and Nucleic Acid Structures, J Mol Biol 2018;430:2244-2248.

7.	Raush E, Totrov M, Marsden BD et al. A new method for publishing three-dimensional content, PLoS One 2009;4:e7394.

8.	Wang J, Youkharibache P, Zhang D et al. iCn3D, a web-based 3D viewer for sharing 1D/2D/3D representations of biomolecular structures, Bioinformatics 2020;36:131-135.

9.	Yiu CB, Chen YW. Molecular Data Visualization on Mobile Devices: A Quick Starter's Guide, Methods Mol Biol 2021;2199:337-346.

10.	Herraez A. Biomolecules in the computer: Jmol to the rescue, Biochem Mol Biol Educ 2006;34:255-261.

11.	Sehnal D, Bittrich S, Deshpande M et al. Mol* Viewer: modern web app for 3D visualization and analysis of large biomolecular structures, Nucleic Acids Res 2021;49:W431-W437.

12.	Norrby M, Grebner C, Eriksson J et al. Molecular Rift: Virtual Reality for Drug Designers, J Chem Inf Model 2015;55:2475-2484.

13.	Cortes Rodriguez F, Dal Peraro M, Abriata LA. Online tools to easily build virtual molecular models for display in augmented and virtual reality on the web, J Mol Graph Model 2022;114:108164.

14.	Bekker GJ, Nakamura H, Kinjo AR. Molmil: a molecular viewer for the PDB and beyond, J Cheminform 2016;8:42.

15.	Berkmen MB, Balla M, Cavanaugh MT et al. An idea to explore: Use of the virtual reality app Nanome for teaching three-dimensional biomolecular structures, Biochem Mol Biol Educ 2025;53:321-329.

16.	Tomasello G, Armenia I, Molla G. The Protein Imager: a full-featured online molecular viewer interface with server-side HQ-rendering capabilities, Bioinformatics 2020;36:2909-2911.

17.	Rigsby RE, Parker AB. Using the PyMOL application to reinforce visual understanding of protein structure, Biochem Mol Biol Educ 2016;44:433-437.

18.	Goddard TD, Ferrin TE. Visualization software for molecular assemblies, Curr Opin Struct Biol 2007;17:587-595.

19.	Sayle RA, Milner-White EJ. RASMOL: biomolecular graphics for all, Trends Biochem Sci 1995;20:374.

20.	Nazipova NN, Shabalina SA, Ogurtsov A et al. SAMSON: a software package for the biopolymer primary structure analysis, Comput Appl Biosci 1995;11:423-426.

21.	Kaplan W, Littlejohn TG. Swiss-PDB Viewer (Deep View), Brief Bioinform 2001;2:195-197.

22.	Meng EC, Goddard TD, Pettersen EF et al. UCSF ChimeraX: Tools for structure building and analysis, Protein Sci 2023;32:e4792.

23.	Martinez X, Baaden M. UnityMol prototype for FAIR sharing of molecular-visualization experiences: from pictures in the cloud to collaborative virtual reality exploration in immersive 3D environments, Acta Crystallogr D Struct Biol 2021;77:746-754.

24.	Skanberg R, Hotz I, Ynnerman A et al. VIAMD: a Software for Visual Interactive Analysis of Molecular Dynamics, J Chem Inf Model 2023;63:7382-7391.

25.	Wang X, Zhuang Y, Liang W et al. VisionMol: a novel virtual reality tool for protein molecular structure visualization and manipulation, Bioinformatics 2025;41.

26.	Humphrey W, Dalke A, Schulten K. VMD: visual molecular dynamics, J Mol Graph 1996;14:33-38, 27-38.

27.	Xu K, Liu N, Xu J et al. VRmol: an integrative web-based virtual reality system to explore macromolecular structure, Bioinformatics 2021;37:1029-1031.

28.	Maria M, Guionniere S, Dacquay N et al. VTX: real-time high-performance molecular structure and dynamics visualization software, Bioinformatics 2025;41.

29.	Krieger E, Vriend G. YASARA View - molecular graphics for all devices - from smartphones to workstations, Bioinformatics 2014;30:2981-2982.
